# Synthesis and Characterization of Isostructural Th(IV) and U(IV) Pyridine Dipyrrolide Complexes

**DOI:** 10.1021/acs.inorgchem.3c04391

**Published:** 2024-02-20

**Authors:** Leyla R. Valerio, Brett M. Hakey, Dylan C. Leary, Erin Stockdale, William W. Brennessel, Carsten Milsmann, Ellen M. Matson

**Affiliations:** † Department of Chemistry, University of Rochester, Rochester, New York 14627, United States; ‡ C. Eugene Bennett Department of Chemistry, West Virginia University, Morgantown, West Virginia 26506, United States

## Abstract

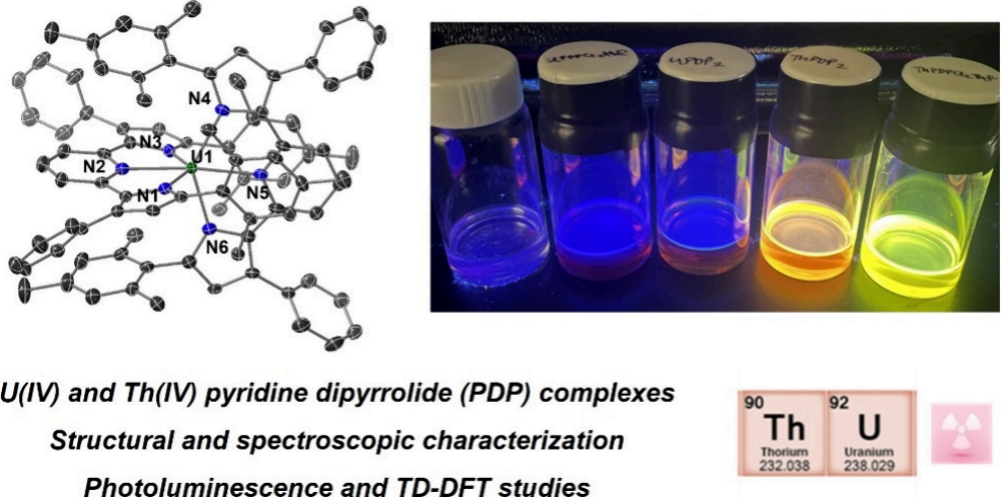

A series of pyridine dipyrrolide actinide(IV) complexes, (^Mes^PDP^Ph^)AnCl_2_(THF) and An(^Mes^PDP^Ph^)_2_ (An = U, Th, where (^Mes^PDP^Ph^) is the doubly deprotonated form of 2,6-bis(5-(2,4,6-trimethylphenyl)-3-phenyl-1*H*-pyrrol-2-yl)pyridine), have been prepared. Characterization of all four complexes has been performed through a combination of solid- and solution-state methods, including elemental analysis, single crystal X-ray diffraction, and electronic absorption and nuclear magnetic resonance spectroscopies. Collectively, these data confirm the formation of the mono- and bis-ligated species. Time-dependent density functional theory has been performed on all four An(IV) complexes, providing insight into the nature of electronic transitions that are observed in the electronic absorption spectra of these compounds. Room temperature, solution-state luminescence of the actinide complexes is presented. Both Th(IV) derivatives exhibit strong photoluminescence; in contrast, the U(IV) species are nonemissive.

## Abstract 2

The synthesis and characterization of isostructural thorium(IV) and uranium(IV) adducts of a pyridine dipyrrolide ligand are reported. The thorium derivatives are photoluminescent, while the uranium complexes are nonemissive.

## Introduction

A fundamental challenge in the study of molecules and materials derived from actinide ions is understanding the electronic structure of these elements.^1−6^ Interest in this area of research is driven, in part, by the lack of clarity in the role that 5f orbitals play in bonding and reactivity. Over the past three decades, progress has been made toward understanding the bonding and reactivity of early actinide complexes with unique ligand sets.^7−9^ It has been demonstrated that the 5f orbitals of the early actinides feature increasing f-orbital participation in bonding across the series (Th ≪ U < Np < Pu). This has been attributed to spin–orbit coupling that results in a decrease in the orbital energy degeneracy and an increase in the energy gaps between the 5f and 6d orbitals.^10,11^ Bonding descriptions of thorium compounds are unique because the 5f orbitals are slightly higher in energy than the 6d orbitals, resulting in bonding dominated by contributions from the metal 6d orbitals and π orbitals of the coordinated ligand. This is a notable difference compared to uranium, neptunium, and plutonium; as such, comparison between the electronic properties of isostructural thorium and uranium complexes presents an opportunity to probe the electronic effects of the addition of increased f-orbital participation in bonding in organoactinide species.

**Figure 1:**
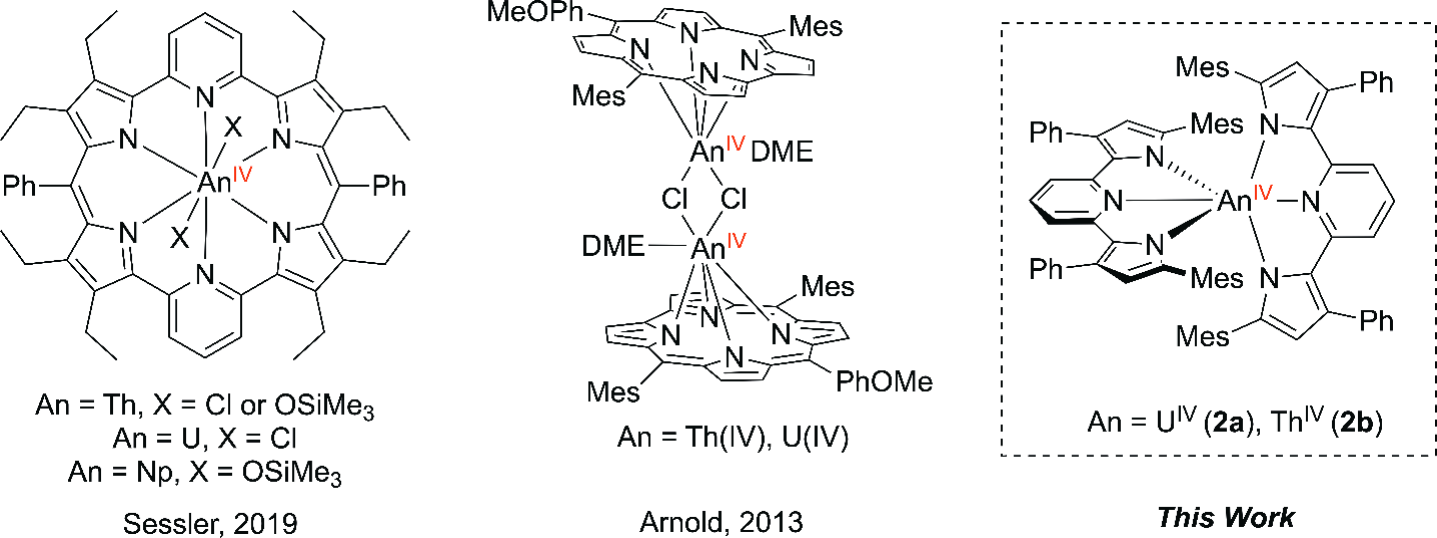
Selected actinide(IV) complexes featuring pyrrole-derived ligands.

Historically, coordination chemistry involving uranium has focused on the uranyl moiety, [UO_2_]^2+^, due to its ubiquity in the environment and nuclear waste. Uranyl complexes have typically possessed ligands stable toward oxygen and moisture, targeting implementation in existing nuclear fuel processes.^12,13^ While pyrrole-derived ligands have been reported widely in studies describing the coordination chemistry of actinyl ions,^14−20^ actinide complexes in the 4+ oxidation state containing these ligands are comparatively scarce (Figure 1). The majority of uranium(IV) and thorium(IV) pyrrole complexes reported in the literature feature polypyrrolic macrocycles that bind uranium and thorium cations.^21−23^ For example, Sessler and co-workers have described a series of Th(IV), U(IV), and Np(IV) complexes featuring coordination to dipyriamethyrin.^21^ Additionally, Arnold and Love have demonstrated that polypyrrolic “Pacman” ligands are excellent platforms for reductively functionalizing uranyl bonds and have recently shown that these macrocycles can also stabilize U^IV^/U^IV^ bent and linear μ-oxo complexes.^22^ Similarly, Arnold and co-workers have reported the utility of a corrole macrocycle, Mes_2_(*p*-OMePh)corrole, to coordinate Th(IV) and U(IV) cations, forming dimeric species bridged via bis(μ-chlorido) linkages.^23^

Of late, a new class of tridentate pincer ligands, pyridine dipyrrolides (PDPs) in the dianionic, doubly deprotonated form, have attracted attention as ligands for both transition metal and main group elements. Interest in these ligands has focused on the utility of the resultant complexes in photochemistry and catalysis.^24−36^ The group(IV)-derived photosensitizer Zr(^Mes^PDP^Ph^)_2_, where (^Mes^PDP^Ph^)^2–^ is the doubly deprotonated form of 2,6-bis(5-(2,4,6-trimethylphenyl)-3-phenyl-1*H*-pyrrol-2-yl)pyridine), was recently reported by some of us.^33^ Thorough photophysical and theoretical studies of this compound have revealed that Zr(^Mes^PDP^Ph^)_2_ possesses a long-lived triplet excited state that exhibits photoluminescence (Φ_PL_ = 0.45). Recent efforts in this space have focused on the isolation of heavier element congeners of the Zr(^Mes^PDP^Ph)^_2_ complex, notably Hf(IV) and Sn(IV), to investigate the effect of heavy atoms on the photophysical properties of the resultant complexes.^38,78^

The precedented coordination chemistry of PDP ligands with heavy group 4 metals, Zr(IV) and Hf(IV) in particular, was the basis of our interest in these ligands for the investigation of Th(IV) and U(IV) coordination chemistry. Indeed, second- and third-row group 4 metals are often considered to be nonradioactive surrogates for Th(IV) and U(IV). The goal of isolating isostructural U(^Mes^PDP^Ph^)_2_ and Th(^Mes^PDP^Ph^)_2_ compounds was identified as a means to probe what consequences the addition of f electrons may have on the electronic properties of the resulting An(^Mes^PDP^Ph^)_2_ complexes. In addition, with the exception of uranyl complexes whose photophysical properties have been studied in depth,^13,37−41^ photophysical studies of molecular actinide compounds are rare.

Herein, we report the synthesis and spectroscopic characterization of a series of pyridine dipyrrolide (PDP) f-element compounds. Access to the targeted bis-ligand species has been enabled through the formation of monoligated, dichloride intermediates, (^Mes^PDP^Ph^)AnCl_2_(THF). Theoretical studies using time-dependent density functional theory have confirmed LMCT transitions for the uranium compounds and a combination of intraligand charge transfer (ILCT) and ligand-to-ligand charge transfer (LLCT) transitions for the thorium congeners. (^Mes^PDP^Ph^)ThCl_2_(THF) and Th(^Mes^PDP^Ph^)_2_ both display strong photoluminescence with quantum yields similar to that of the Zr analogue and long lifetimes on the order of microseconds.

## Experimental Section

### General Considerations

All air- and moisture-sensitive manipulations were carried out using a standard high-vacuum line, Schlenk techniques, or an MBraun inert atmosphere drybox containing an atmosphere of purified dinitrogen. All solids were dried under high vacuum to bring into the glovebox. Solvents for air- and moisture-sensitive manipulations were dried and deoxygenated using a glass contour solvent purification system (Pure Process Technology, LLC) and stored over activated 4 Å molecular sieves (Fisher Scientific) prior to use. Deuterated solvents for ^1^H NMR spectroscopy were purchased from Cambridge Isotope Laboratories and stored in the glovebox over activated 3 Å molecular sieves after three freeze–pump–thaw cycles. Chemicals were purchased from commercial sources and used without further purification. UCl_4_, ThCl_4_(DME)_2_, H_2_^Mes^PDP^Ph^, and K(CH_2_Ph) were synthesized following reported procedures.^27,42−44^

### Safety Considerations

***Caution!** Depleted uranium (primary isotope ^238^U) is a weak α-emitter (4.197 MeV) with a half-life of 4.47 × 10^9^ years, and ^232^Th is a weak α-emitter (4.082 MeV) with a half-life of 1.41 × 10^10^ years; manipulations and reactions should be carried out in monitored fume hoods or in an inert atmosphere drybox in a radiation laboratory equipped with α and β counting equipment.*

#### Synthesis of (^Mes^PDP^Ph^)MCl_2_(THF); M = U (**1a**), Th (**1b**)

In the glovebox, H_2_^Mes^PDP^Ph^ (0.015 g, 0.025 mmol, 1 equiv) was dissolved in approximately 3 mL of THF in a 20 mL scintillation vial equipped with a stir bar. In a separate vial, LiN(SiMe_3_)_2_ (0.009 g, 0.053 mmol, 2.1 equiv) was dissolved in 3 mL of THF and added dropwise to the stirring H_2_^Mes^PDP^Ph^ solution to make Li_2_^Mes^PDP^Ph^. The resultant solution was stirred for 2 h and then transferred to a 15 mL pressure vessel equipped with a stir bar. The actinide metal salt, UCl_4_ or ThCl_4_(DME)_2_ (0.01 g, 1 equiv), was dissolved in THF and added dropwise to the Li_2_^Mes^PDP^Ph^ solution while stirring. The pressure vessel was sealed with a Teflon-lined cap, removed from the glovebox, and stirred overnight at 65 °C. The next day, the pressure vessel was cooled to room temperature and brought back into the glovebox. The resulting red (U) or yellow (Th) solution was transferred to a vial and dried under vacuum. The solid was triturated with pentane until washings ran clear and was dried under reduced pressure. The solid was dissolved in benzene and filtered over Celite and a glass frit, followed by removal of the solvent *in vacuo*, affording the title compound.

#### (^Mes^PDP^Ph^)UCl_2_(THF) (**1a**)

(0.015 g, 0.015 mmol, 62%.) ^1^H NMR (400 MHz, C_6_D_6_) δ 9.75 (s, 12H), 3.94 (d, *J* = 7.4 Hz, 2H), 3.81 (t, *J* = 7.8 Hz, 4H), −0.13 (d, *J* = 8.4 Hz, 4H), −1.57 (s, 4H), −2.79 (s, 2H), −4.75 (s, 1H), −5.59 (s, 6H), −13.68 (s, 2H), −20.61 (s, 4H), −29.91 (s, 4H). Crystals suitable for single crystal X-ray diffraction were grown from the slow diffusion of pentane into a toluene solution of the compound at −30 °C. Anal. Calcd for C_47_H_45_Cl_2_N_3_OU (mol. wt. 976.824 g/mol): C, 57.79%; H, 4.64%; N, 4.30%. Found: C, 57.64%; H, 4.63%; N, 3.99%.

#### (^Mes^PDP^Ph^)ThCl_2_(THF) (**1b**)

(0.008 g, 0.008 mmol, 47%.) ^1^H NMR (400 MHz, C_6_D_6_) δ 7.56–7.51 (m, 4H), 7.19 (s, 6H), 7.05 (d, *J* = 8.1 Hz, 2H), 6.69 (s, 4H), 6.43 (t, *J* = 8.1 Hz, 1H), 6.15 (s, 2H), 2.58 (s, 12H), 2.01 (s, 4H), 1.28–1.23 (m, 4H). ^13^C NMR (101 MHz, C_6_D_6_) δ 155.74, 141.32, 140.34, 139.95, 138.75, 138.04, 137.88, 136.04, 130.71, 130.27, 128.69, 128.64, 127.00, 114.48, 113.77, 25.51, 21.66, 21.10. Crystals suitable for single crystal X-ray diffraction were grown from the slow diffusion of pentane into a toluene solution of the compound at −30 °C. Anal. Calcd for C_47_H_45_Cl_2_N_3_OTh (mol. wt. 970.835 g/mol): C, 58.15%; H, 4.67%; N, 4.33%. Found: C, 58.15%; H, 4.59%; N, 4.19%.

#### Synthesis of M(^Mes^PDP^Ph^)_2_; M = U (**2a**), Th (**2b**)

In a 20 mL scintillation vial, (^Mes^PDP^Ph^)MCl_2_(THF) (U: 0.05 g, 0.051 mmol; Th: 0.028 g, 0.029 mmol) was dissolved in a minimal amount of THF. In a separate vial, K(CH_2_Ph) (2.1 equiv) was dissolved in THF, and both vials were frozen completely in the glovebox coldwell at −80 °C. Upon thawing, the (^Mes^PDP^Ph^)MCl_2_(THF) solution was added to the second vial, inducing a color change to dark burgundy (U) or dark red (Th). The vial was immediately dried under vacuum. The solid was suspended in diethyl ether and filtered over a glass frit with Celite. The resultant solution was pumped dry to afford the intermediate compound (^Mes^PDP^Ph^)M(CH_2_Ph)_2_(THF) (U: 0.028 g, 0.026 mmol; Th: 0.024 g, 0.022 mmol). This intermediate complex was dissolved in toluene and transferred to a 15 mL pressure vessel equipped with a stir bar. H_2_^Mes^PDP^Ph^ (U: 0.014 g, 0.023 mmol; Th: 0.013 g, 0.022 mmol) was suspended in toluene and added to the pressure vessel, followed by sealing the vessel with a Teflon-lined cap. The pressure vessel was brought out of the glovebox, set to stir, and heated to 120 °C for 2 days. Then the dark-red (U) or bright-orange (Th) solution was allowed to cool to room temperature before being brought back into the glovebox. The resulting product was filtered over a glass frit packed with Celite and dried *in vacuo*. The solid was triturated with pentane until washings ran clear and was dried again to afford the title compound.

#### U(^Mes^PDP^Ph^)_2_ (**2a**)

(0.025 g, 0.017 mmol, 69%.) ^1^H NMR (400 MHz, C_6_D_6_) δ 18.47 (s, 4H), 12.28 (s, 24H), 6.21 (t, *J* = 7.8 Hz, 8H), 6.07–5.98 (m, 12H), 2.89 (s, 8H), −2.87 (s, 12H), −3.44 (t, *J* = 7.7 Hz, 2H), −8.42 (d, *J* = 8.1 Hz, 4H). Crystals suitable for single crystal X-ray diffraction were grown from a concentrated diethyl ether solution of the compound at −30 °C. Anal. Calcd for C_86_H_74_N_6_U (mol. wt. 1429.65 g/mol): C, 71.25%; H, 5.22%; N, 5.88%. Found: C, 71.37%; H, 5.22%; N, 5.53%.

#### Th(^Mes^PDP^Ph^)_2_ (**2b**)

(0.023 g, 0.023 mmol, 77%.) ^1^H NMR (500 MHz, C_6_D_6_) δ 7.70 (d, *J* = 7.5 Hz, 8H), 7.35 (q, *J* = 7.7 Hz, 8H), 7.21 (t, *J* = 7.6 Hz, 8H), 6.94 (d, *J* = 8.1 Hz, 4H), 6.54 (d, *J* = 15.7 Hz, 11H), 6.05 (s, 4H), 2.16 (s, 24H), 1.94 (s, 12H). ^13^C NMR (101 MHz, C_6_D_6_) δ 154.85, 141.59, 139.14, 138.77, 136.93, 135.97, 130.98, 130.35, 130.20, 128.73, 126.99, 116.62, 113.45, 22.25, 21.09, 20.50. Crystals suitable for single crystal X-ray diffraction were grown from a concentrated diethyl ether solution of the compound at −30 °C. Anal. Calcd for C_86_H_74_N_6_Th (mol. wt. 1423.66 g/mol): C, 71.08%; H, 5.25%; N, 5.72%. Found: C, 71.06%; H, 5.11%; N, 5.33%.

### Physical Measurements

^1^H NMR spectra were recorded at room temperature on a 400 MHz Bruker AVANCE spectrometer or a 500 MHz Bruker AVANCE spectrometer locked on the signal of deuterated solvents. All of the chemical shifts are reported relative to the chosen deuterated solvent as a standard. Electronic absorption measurements were recorded at room temperature in anhydrous toluene in sealed 1 cm quartz cuvettes using an Agilent Cary 6000i UV–vis–NIR spectrophotometer. Emission spectra were collected with a Spex Fluoromax-3 fluorometer (Horiba) with a photomultiplier tube detector. Sample absorbances were kept below 0.2 OD in anhydrous toluene in 1 cm quartz cuvettes. Time-correlated single photon counting (TCSPC) measurements were acquired using a home-built optical setup. Samples in anhydrous toluene solution were placed in 1 cm quartz cuvettes and photoexcited by a defocused laser beam provided by a pulsed laser diode (PicoHarp 300, PDL 800-D). Data was collected on a Tektronix TBS 1102B-Edu digital oscilloscope. Elemental analysis data were obtained from the Elemental Analysis Facility at the University of Rochester. Microanalysis samples were weighed with a PerkinElmer model AD6000 autobalance, and their compositions were determined with a PerkinElmer 2400 series II analyzer. Air-sensitive samples were handled in a VAC Atmospheres glovebox.

### X-ray Crystallography

Each single crystal was collected on a nylon loop and mounted on a Rigaku XtaLAB Synergy-S Dualflex diffractometer equipped with a HyPix-6000HE HPC area detector for data collection at 100.00(10) K. A preliminary set of cell constants and an orientation matrix were calculated from a small sampling of reflections.^45^ A short pre-experiment was run, from which an optimal data collection strategy was determined. The full data collection for all four complexes was carried out using a PhotonJet (Cu) X-ray source. After the intensity data were corrected for absorption, the final cell constants were calculated from the xyz centroids of the strong reflections from the actual data collections after integration.^45^ The structure was solved using SHELXT^46^ and refined using SHELXL.^47^ Most or all non-hydrogen atoms were assigned from the solution. Full-matrix least-squares/difference Fourier cycles were performed, which located any remaining non-hydrogen atoms. All of the non-hydrogen atoms were refined with anisotropic displacement parameters. All of the hydrogen atoms were placed in ideal positions and refined as riding atoms with relative isotropic displacement parameters. Structures **1a** and **1b** are isomorphous; structures **2a** and **2b** are as well.

For complexes **1a** and **1b**, the asymmetric unit contains one metal complex in a general position and one-half each of three toluene solvent molecules in special positions. Toluene molecules C48-C51 and C52-C55 are modeled as disordered over crystallographic 2-fold axes (0.50:0.50), and toluene molecule C56-C62 is modeled as disordered over a crystallographic inversion center (0.50:0.50). For complexes **2a** and **2b**, the asymmetric unit contains two metal complexes in general positions and one-half of a diethyl ether solvent molecule on a crystallographic inversion center; the ether molecule is modeled as disordered over the inversion center (0.50:0.50).

### Computational Methods

All calculations were performed using the ORCA quantum chemical program package, version 5.0.1.^48,49^ Geometry optimizations used the PBE functional^50^ and were accelerated using the resolution of identity (RI) approximation.^51,52^ Scalar-relativistic effects were included via the zeroth-order regular approximation (ZORA)^53^ using the relativistically recontracted triple-ζ quality basis set ZORA-def2-TZVP^54^ on nitrogen, oxygen, and chlorine atoms and SARC-ZORA-TZVP^55^ on thorium and uranium. All other atoms were handled with the recontracted split-valence ZORA-def2-SVP basis set.^54^ Noncovalent interactions were considered via atom-pairwise dispersion corrections with Becke–Johnson (D3BJ) damping.^56,57^ The TD-DFT calculations used the B3LYP density functional^58^ and were accelerated using the RIJCOSX approximation.^59,60^ Relativistic effects were included using the Douglas–Kroll–Hess (DKH) Hamiltonian with DKH-specific basis sets analogous to those used in the geometry optimizations (SARC-DKH-TZVP, DKH-def2-TZVP, and DKH-def2-SVP). The Tamm–Dancoff approximation was not used, and the effects of spin–orbit coupling (SOC) were probed using a spin–orbit mean field (SOMF) approach.^61^ All solvation effects were handled using the conductor-like polarizable continuum model (C-PCM) and a Gaussian charge scheme.^62^ All orbital and spin-density plots were generated using the program Gabedit.^63^

## Results and Discussion

### Synthesis and Characterization of (^Mes^PDP^Ph^)UCl_2_(THF) Complexes

Initial attempts to synthesize An^IV^(^Mes^PDP^Ph^)_2_ (An = Th, U) complexes focused on the uranium(IV) derivative. Two equivalents of LiN(SiMe_3_)_2_ were added to a solution of H_2_^Mes^PDP^Ph^ in THF, affording the *in situ* generation of Li_2_^Mes^PDP^Ph^. Addition of this solution to 0.5 equiv of UCl_4_ in THF resulted in a gradual color change to dark red; after stirring the reaction mixture overnight, volatiles were removed under reduced pressure. Analysis of the crude reaction mixture by ^1^H NMR spectroscopy revealed the formation of a major product featuring 11 paramagnetically shifted and broadened resonances (Figure S1), with a general pattern resembling that of the monoligated uranium(IV) siloxide complex, (^Mes^PDP^Ph^)U(OSiMe_3_)_2_(DMAP) (where DMAP = 4-dimethylaminopyridine), reported previously by our group.^64^ Free ligand was also observed in the ^1^H NMR spectrum of the crude product. Collectively, these results suggest that only a single PDP ligand is added to the uranium center under the described reaction conditions.

**Scheme 1:**
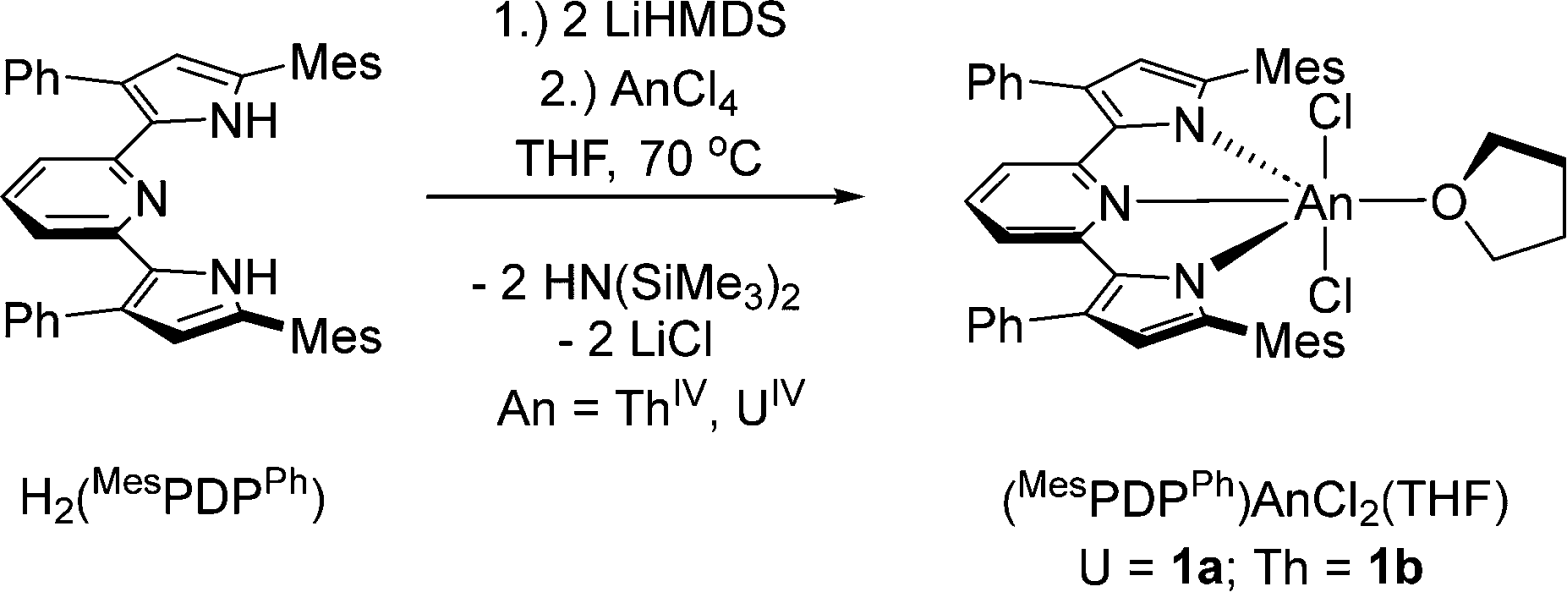
Synthesis of (^Mes^PDP^Ph^)AnCl_2_(THF) Complexes (An = U (**1a**); Th (**1b**))

Further support for addition of a single ligand to the uranium center was obtained through targeted synthesis of the monoligated complex, (^Mes^PDP^Ph^)UCl_2_(THF). The addition of 1 equiv of Li_2_^Mes^PDP^Ph^ to a solution of UCl_4_ in THF results in a gradual color change to dark red after heating to 70 °C for 16 h. Workup of the product afforded (^Mes^PDP^Ph^)UCl_2_(THF) (**1a**) in 60% yield (Scheme 1; see Experimental Section for additional details). The formation of the monoligated uranium species was confirmed by ^1^H NMR spectroscopy; 11 paramagnetically shifted and broadened resonances ranging from +10 to −30 ppm were observed (Figure S2), matching the result obtained in the aforementioned attempt to access U^IV^(^Mes^PDP^Ph^)_2_. The distribution of resonances in the ^1^H NMR spectrum is consistent with the formation of a product with *C*_*2v*_ symmetry. Closer inspection of the integrations in the ^1^H NMR spectrum of (^Mes^PDP^Ph^)UCl_2_(THF) reveals a resonance at −4.75 ppm with a relative integration of one and is assigned to the 4-pyridyl proton. This particular signal is typically used as a spectroscopic benchmark for PDP ligands bound to transition metal and metalloid centers.^24,65^ Moreover, a diagnostic singlet is observed at −2.79 ppm and is attributed to the 4-pyrrolide hydrogens. The highest-intensity signal in the spectrum is located at 9.75 ppm, with a relative integration of 12. We assign this resonance to the *ortho*-methyl groups of the mesityl substituent of the PDP ligand. A signal located at −5.59 ppm with a relative integration of six is assigned to the corresponding *para*-methyl group protons of the mesityl functionality.

**Figure 2:**
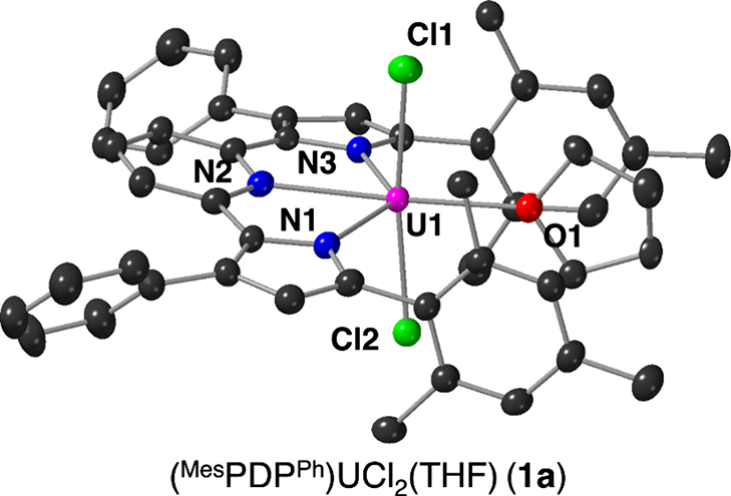
Molecular structure of (^Mes^PDP^Ph^)UCl_2_(THF) (**1a**) shown with 30% probability ellipsoids. The molecular structure of (^Mes^PDP^Ph^)ThCl_2_(THF) (**1b**) is similar to that of **1a**; the corresponding image can be found in the Supporting Information (Figure S4). Hydrogen atoms and solvent molecules have been removed for clarity. Key: pink, U; blue, N; gray, C; light green, Cl; red, O.

Crystals of **1a** suitable for single crystal X-ray diffraction (SCXRD) were grown from the slow diffusion of pentane into a concentrated solution of the product in toluene at −30 °C. Refinement of the data confirmed the structural composition of **1a** as the anticipated six-coordinate species (^Mes^PDP^Ph^)UCl_2_(THF) (Figure 2, Table 1). Complex **1a** displays a distorted octahedral geometry at the uranium center; the equatorial positions of the octahedron are defined by the meridionally coordinated N_3_-(^Mes^PDP^Ph^) chelate and the tetrahydrofuran ligand. Two chloride ligands occupy the axial sites, and the pincer ligand enforces a N1–U–N3 bite angle of 132.67(16)° for **1a**. This structural feature represents the largest deviation from octahedral geometry in **1a**. The U–N_pyridine_ distance of 2.474(2) Å is significantly shortened in comparison to that of (^Mes^PDP^Ph^)U(OSiMe_3_)_2_(DMAP) (U–N_pyridine_ = 2.545(3) Å).^64^ Truncation of the U–N_pyrrolide_ distances (U–N1, U–N3) of **1a** (U–N_pyrrolide_ = 2.309(5), 2.301(5) Å) is also observed in comparison to (^Mes^PDP^Ph^)U(OSiMe_3_)_2_(DMAP) (U–N_pyrrolide_ = 2.545(3) Å). The U–N_pyrrolide_ distances are among the shortest reported for U(IV)-pyrrolide species (U(IV)–N_pyrrolide_ = 2.389(3)–2.531(7) Å).^66−68^ Collectively, these results suggest that the uranium(IV) center binds more tightly to the (^Mes^PDP^Ph^)^2–^ ligand upon substitution of the electron-donating siloxide moieties with chlorido ligands. The U–Cl bond distances in **1a** (2.5844(13) and 2.5927(14) Å) are slightly shorter than values reported previously for U(IV)Cl_2_(L) (L = neutral ligand) complexes (2.614(2)–2.751(3) Å).^21,69−71^ We hypothesize that the *trans* positioning of the chloride substituents renders the inverse *trans* influence operative in these axially bound X-type ligands.^72^

**Table 1:** Pertinent Bond Distances and Angles for Complexes (^Mes^PDP^Ph^)AnCl_2_(THF) (An = U, **1a**; Th, **1b**), with Distances and Angles for (^Mes^PDP^Ph^)U(OSiMe_3_)_2_(DMAP)^64^ Included for Comparison

**Complex**	**(**^**Mes**^**PDP**^**Ph**^**)UCl**_**2**_**(THF) (1a)** An = U, E = Cl	**(**^**Mes**^**PDP**^**Ph**^**)ThCl**_**2**_**(THF) (1b)** An = Th, E = Cl	**(**^**Mes**^**PDP**^**Ph**^**)U(OSiMe**_**3**_**)**_**2**_**(DMAP)** An = U, E = OSiMe_3_
An–E	2.5844(13), 2.5927(14) Å	2.6528(10), 2.6566(11) Å	2.114(2), 2.123(2) Å
E–An–E	174.04(4)°	172.33(3)°	178.95(8)°
An–N_pyr_	2.474(4) Å	2.548(3) Å	2.545(3) Å
An–N_pyrrolide_	2.301(5), 2.309(5) Å	2.362(3), 2.358(4) Å	2.369(3), 2.371(3) Å

With complex **1a** in hand, we next targeted the preparation of the isostructural thorium analogue, (^Mes^PDP^Ph^)ThCl_2_(THF) (**1b**). The synthesis of **1b** is motivated by our interest in comparing the electronic structure of actinide complexes with and without 5f electrons (U(IV) = 5f^2^ vs Th(IV) = 5f^0^). The addition of *in situ*-generated Li_2_^Mes^PDP^Ph^ in THF to a solution of ThCl_4_(DME)_2_ in the same solvent resulted in a gradual color change to yellow after heating to 70 °C for 16 h. Workup of the solution afforded complex **1b** in 46% yield (Scheme 1). Characterization of the purified product by ^1^H NMR spectroscopy reveals the expected number of resonances (11) with relative integrations for a *C*_*2v*_-symmetric product in solution (Figure S3). Characteristic signals measured in the ^1^H NMR spectrum of **1b** include a triplet at 6.43 ppm assigned to the 4-pyridyl proton. A diagnostic singlet is also observed at 6.14 ppm, corresponding to the 4-pyrrolide hydrogens. The *ortho*- and *para*-methyl groups of the mesityl moiety on the PDP ligand are located at 2.58 and 2.10 ppm, respectively, with relative integrations of 12:6. Resonances assigned to a bound THF ligand were identified at 3.37 (α-CH_2_) and 1.10 (β-CH_2_) ppm for **1b**, with integrations consistent with a 1:1 THF:(^Mes^PDP^Ph^) ligand ratio. ^13^C NMR of **1b** was also obtained and is consistent with the anticipated spectrum of the product (Figure S5).

Yellow crystals of (^Mes^PDP^Ph^)ThCl_2_(THF) suitable for analysis via SCXRD were obtained from the slow diffusion of pentane into a concentrated toluene solution of **1b**. Refinement of the data confirmed the identity of complex **1b** as the pseudo-octahedral, six-coordinate species (^Mes^PDP^Ph^)ThCl_2_(THF) (Figure 2, Table 1). Notably, complexes **1a** and **1b** both crystallize in the *Pbcn* space group and with nearly identical unit cells. As observed in **1a**, the (^Mes^PDP^Ph^)^2–^ ligand and a bound tetrahydrofuran molecule occupy the equatorial plane of **1b**, with two chloride atoms observed in the axial positions in a *trans* configuration. The Th–N_pyrrolide_ distances of 2.362(3) and 2.358(4) Å are significantly shorter than values reported for a similar thorium(IV) bischloride complex bound to the tripyrrolide dianionic ligand, [(2,5-[(C_4_H_3_N)CPh_2_]_2_[C_4_H_2_N(Me))]ThCl_2_(thf) (Th–N_pyrrolide_ = 2.408(7) and 2.399(7) Å).^73^ This feature of the molecular structure is reminiscent of complex **1a**, which also displays truncated An–N_pyrrolide_ bonds. The Th–Cl bond lengths in (^Mes^PDP^Ph^)ThCl_2_(THF) (2.6528(10), 2.6566(11) Å) are similar to values reported for [(C_4_H_3_N)CPh_2_]_2_[C_4_H_2_N(Me)]ThCl_2_(THF) (2.651(2), 2.681(2) Å) and also fall in a similar range as bound chloride atoms in a thorium complex with a 4,5-bis(2,6-diisopropylanilino)-2,7-di-*tert*-butyl-9,9-dimethylxanthene ligand (Th–N_amido_ = 2.698(3), 2.686(3) Å).^3^

**Figure 3:**
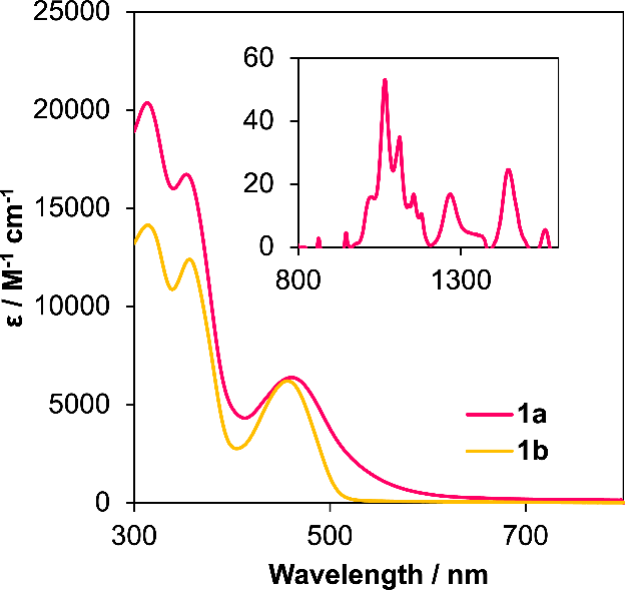
Electronic absorption spectra for (^Mes^PDP^Ph^)UCl_2_(THF) (**1a**) and (^Mes^PDP^Ph^)ThCl_2_(THF) (**1b**) collected at room temperature in toluene. The inset shows the near-infrared region of the electronic absorption spectrum of **1a** to highlight f–f transitions of the U(IV) center.

The striking differences in color between the uranium (dark red) and thorium (bright yellow) derivatives of (^Mes^PDP^Ph^)AnCl_2_(THF) prompted further characterization by electronic absorption spectroscopy. To our surprise, the electronic absorption spectra of **1a** and **1b** in toluene are quite similar (Figure 3). The UV region of each spectrum is dominated by two absorption features that are reminiscent of the absorption maxima of the free ligand, H_2_^Mes^PDP^Ph^ (364 and 316 nm in THF), and therefore were assigned to originate predominantly from π–π* transitions within the individual ligands. Notably, these absorption bands are slightly shifted from those of the protonated PDP ligand, consistent with successful metalation (Figure S6); for an in-depth discussion of the absorption profile of the ligand, we direct readers to a recent report from Milsmann and co-workers.^74^ In the electronic absorption spectrum of **1b**, these two intense bands are observed at 318 nm (ε = 14,006 M^–1^ cm^–1–^) and 360 nm (ε = 12,215 M^–1^ cm^–1^). In addition, complex **1b** also contains a lower-energy transition at 458 nm (ε = 6,203 M^–1^ cm^–1–^), distinct from the electronic absorbance spectrum of H_2_^Mes^PDP^Ph^. A more detailed analysis of this low-energy feature is provided in the computational section below.

In the case of the uranium complex, the electronic absorbance spectrum of **1a** features two intense bands at 316 nm (ε = 20,273 M^–1^ cm^–1–^) and 356 nm (ε = 16,650 M^–1^ cm^–1–^). Similar to **1b**, complex **1a** also contains a lower-energy feature at 462 nm (ε = 6,391 M^–1^ cm^–1^). However, the band shape of this absorption event is quite different than that observed in the case of **1b**. A significant increase in bandwidth results in an extension of absorption to ∼600 nm, translating to the dark-red color observed visually for complex **1a**. This unique feature is reminiscent of a ligand-to-metal charge transfer (LMCT) transition measured experimentally and confirmed by time-dependent density functional theory (TD-DFT) in (^Mes^PDP^Ph^)UO_2_(THF).^65^ An analysis of the near-infrared region of the electronic absorbance spectrum of **1a** also revealed weak and sharp f–f transitions, consistent with the assignment of a +4 oxidation state (5f^2^ valence electron configuration) of uranium.

To gain insight into the origin of the observed electronic transitions in the electronic absorption spectra of **1a** and **1b**, density functional theory (DFT) and time-dependent DFT (TD-DFT) calculations were performed on (^Mes^PDP^Ph^)UCl_2_(THF) and (^Mes^PDP^Ph^)ThCl_2_(THF). Full molecule geometry optimizations were performed at the PBE/D3BJ level of theory. Relativistic effects due to the actinide ions were included through use of the zeroth-order regular approximation (ZORA) with the appropriate relativistically recontracted basis sets. The resulting structural parameters for **1a** and **1b** are in good agreement with the experimental data. Single-point calculations at the B3LYP level of theory using the Douglas–Kroll–Hess (DKH) formalism to include relativistic effects were then performed to obtain insights into the molecular orbital manifolds of **1a** and **1b**.

Based on the experimentally observed diamagnetism of thorium derivative **1b**, a singlet ground state was assumed for all calculations and provided an electronic structure consistent with a +IV oxidation state for the thorium center with seven unoccupied f orbitals. Consistent with largely ionic metal–ligand interactions, the filled frontier molecular orbitals show negligible contributions from the thorium ion with less than 5% thorium character in the HOMO to HOMO–17. The HOMO of **1b** is exclusively ligand-centered with major contributions from the π-systems of the electron-rich pyrrolide heterocycles, resembling the HOMOs of the ligand precursor, H_2_^Mes^PDP^Ph^, and its lithium salt, Li_2_^Mes^PDP^Ph^.^75^ Similarly, the LUMO and LUMO+1 are predominantly ligand-centered with major contributions from the pyridine π system and only minor contributions from thorium 6d orbitals (LUMO: 11% Th character, LUMO+1: 8% Th character). Like the HOMO, the LUMO of **1b** resembles those of H_2_^Mes^PDP^Ph^ and Li_2_^Mes^PDP^Ph^, providing a first indication that the lowest-energy excited state in **1b** should have intraligand character (IL).

**Figure 4:**
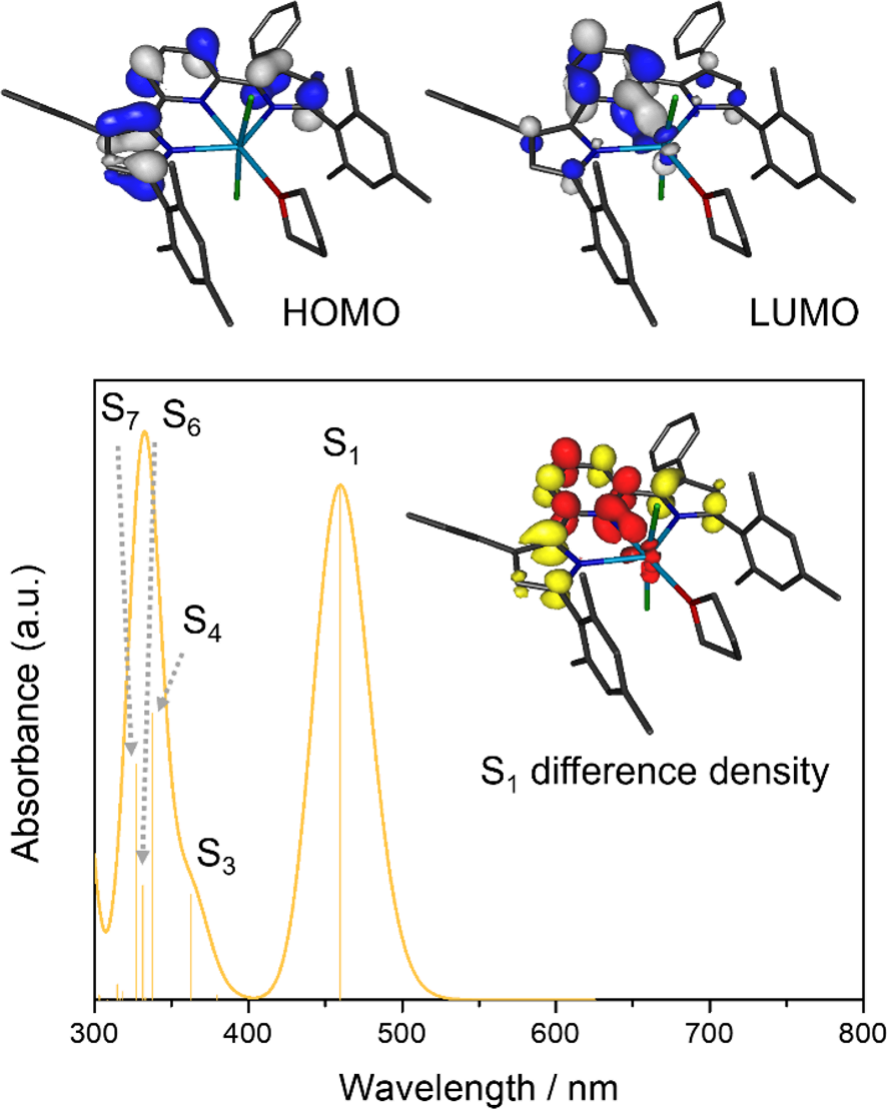
Top: HOMO and LUMO of (^Mes^PDP^Ph^)ThCl_2_(THF) obtained by DFT calculations. Bottom: TD-DFT calculated absorption spectrum (fwhm = 2000 cm^–1^) with individual transitions indicated by the stick plot. The inset shows the difference density for the lowest-energy excited state (red = increased electron density, yellow = reduced electron density), highlighting its ^1^IL character.

To further support our electronic structure analysis, TD-DFT calculations were performed at the B3LYP/DKH level of theory. The computed electronic absorption spectrum is in good agreement with the experimental data and exhibits a lowest-energy absorption band at 460 nm (Figure 4). Further analysis of the computational data revealed that this spectral feature can be attributed to a transition to the S_1_ excited state that is best described as the result of a dipole-allowed single-electron excitation from the HOMO to the LUMO of **1b**. The dominating intraligand character of the S_1_ state (^1^IL), implied by the molecular orbital composition of the HOMO and LUMO, is further supported by Mulliken population analysis for the S_1_ state that reveals only minor charge migration from the ligand to the metal (Δ*q*_Th_ = −0.12 e; Figure 4) compared to the ground state. Four additional transitions with appreciable intensities contribute to the second absorption feature predicted in the UV region between 300 and 400 nm. These are assigned as transitions to the S_3_, S_4_, S_6_, and S_7_ states and are all predominantly ^1^IL in nature. The absence of any low-energy ligand-to-metal charge transfer (LMCT) transitions is consistent with the very negative reduction potential for Th(IV) that indicates that occupation of the metal f and d orbitals is unfavorable for thorium.^75^

**Figure 5:**
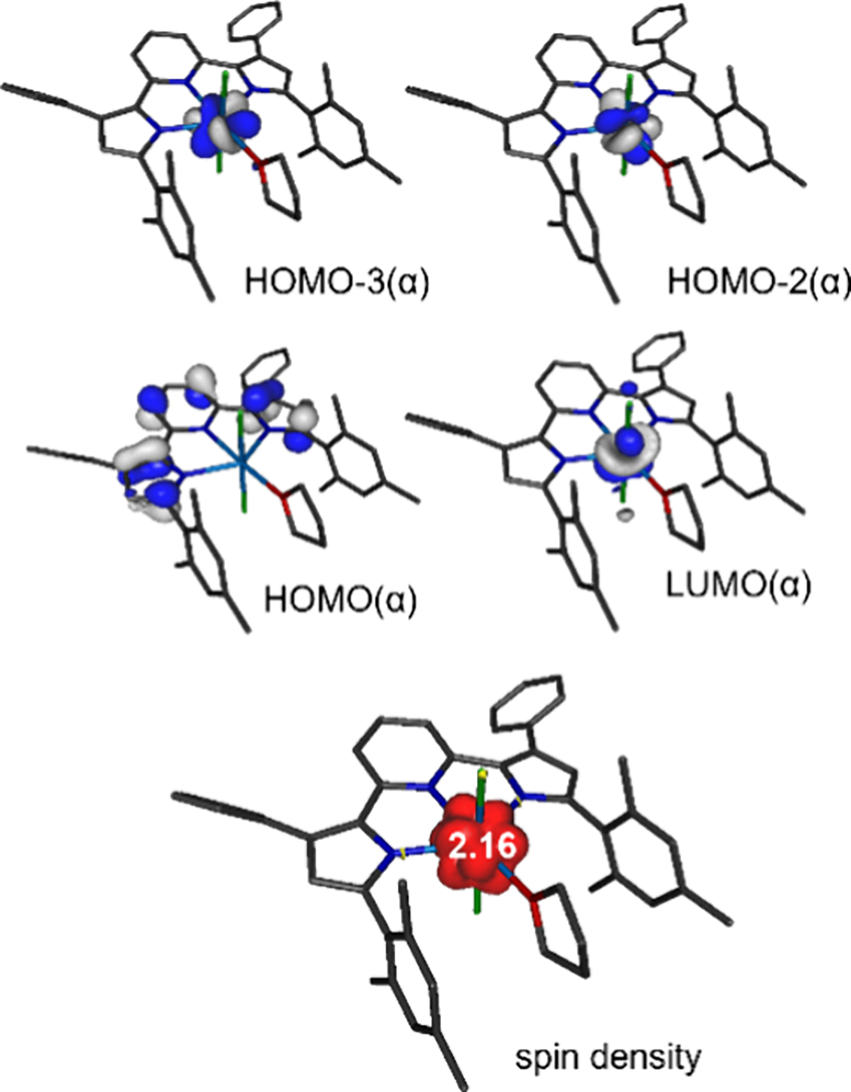
Top: Selected frontier molecular orbitals of (^Mes^PDP^Ph^)UCl_2_(THF) obtained by DFT calculations. The HOMO–2(α) and HOMO–3(α) have no corresponding occupied analogs in the β-spin orbital manifold and represent the magnetic orbitals (SOMOs) of the complex. Bottom: Spin density obtained by Mulliken population analysis for (^Mes^PDP^Ph^)UCl_2_(THF).

A more complex electronic structure analysis, using the unrestricted Kohn–Sham (UKS) formalism, is required for the paramagnetic uranium complex, **1a**. Considering the experimentally assigned U(IV) oxidation state (5f^2^) of the complex, a triplet ground state was assumed for all calculations, and the computational results for **1a** are consistent with a +IV oxidation state of the central actinide ion (Figure 5). As expected for a 5f^2^ configuration, two electrons are located in α-spin orbitals with majority contributions from uranium 5f orbitals (HOMO–3(α) 74% and HOMO–2(α) 58% U character), while all uranium 5f orbitals are unoccupied for the β-spin manifold. Mulliken population analysis provides a spin density value of 2.16 for uranium, also consistent with a 5f^2^ configuration and a triplet ground state. For both the α and β sets of orbitals of **1a**, the HOMO and HOMO–1 are almost identical to those of thorium analog **1b** and are exclusively ligand-centered (0% U character, major contributions from the PDP π system). In contrast, the LUMO(α) is best described as a uranium 5f orbital (79% U contribution), while the LUMO(β) shows more limited yet still substantial metal 5f character (39%). Therefore, low-energy transitions with significant LMCT character can be expected for **1a** in contrast to **1b**, consistent with the more moderate potentials for the U^IV^/U^III^ redox couple.^75^

The paramagnetic nature and the presence of low-energy f–f excited states also complicate the computational analysis of the electronic transitions for **1a** compared to **1b**. An accurate prediction of such metal-centered transitions and any potential charge transfer transitions involving the metal center would require the use of high-level multireference *ab initio* methods with large active spaces and is beyond the scope of this study. Instead, TD-DFT calculations were utilized to probe the presence of low-energy charge transfer states. While this approach cannot be expected to yield quantitatively accurate energies for the resulting electronic transitions or an accurate reproduction of the experimental electronic absorption spectrum overall, it nevertheless provides qualitative insight into the accessibility of charge transfer states in **1a** in comparison to **1b**.

**Figure 6:**
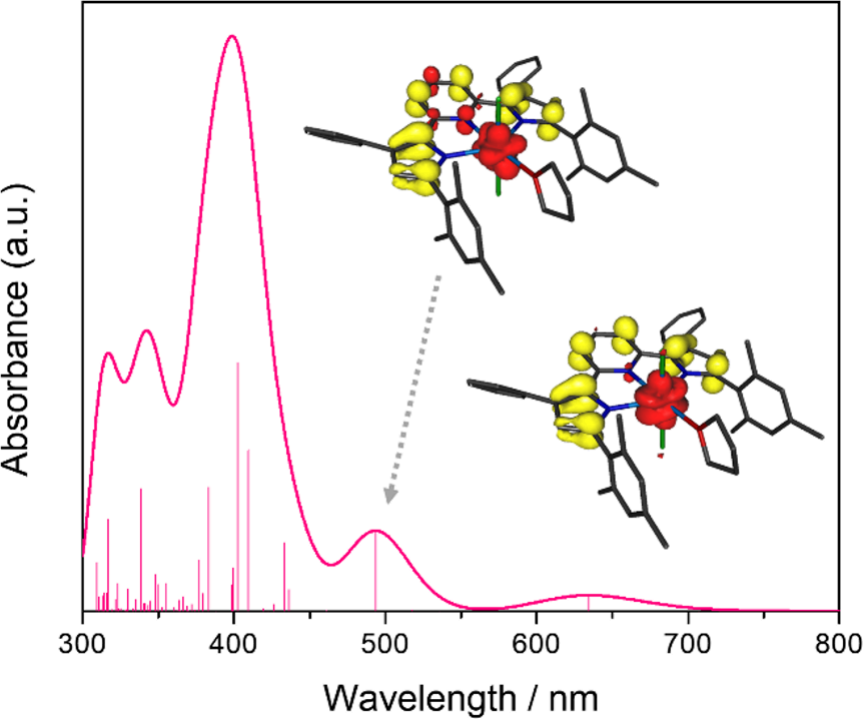
TD-DFT calculated absorption spectrum (fwhm = 2000 cm^–1^) including the first 70 transitions for (^Mes^PDP^Ph^)UCl_2_(THF) with individual transitions indicated by the stick plot. The insets show the difference densities for the two lowest-energy charge transfer excited states (red = increased electron density, yellow = reduced electron density), highlighting their ^1^LMCT character.

Within the first 70 TD-DFT excitations, the lowest-energy transition excluding f–f excited states was identified as an absorption band at 634 nm. The corresponding excited state has significant ^3^LMCT character as reflected in the significant charge migration from the PDP ligand to the uranium center (Δ*q*_U_ = −0.74 e). This charge distribution can be understood by an inspection of the unrelaxed difference density for this transition (Figure 6); a significant loss of electron density for the pyrrolide rings and an increase in electron density at the uranium ion are observed. Further supporting LMCT character, the spin density at the metal center increases to 2.83, in line with an f^3^ configuration and three unpaired electrons for uranium in this excited state. A second, more intense absorption feature centered at 494 nm can also be attributed to a single excited state with significant ^3^LMCT character (Δ*q*_U_ = −0.32 e). The most intense absorption feature within the first 70 TD-DFT states was observed at 399 nm and is a combination of several energetically close-lying excited states that are predominantly IL in character (Δ*q*_U_ < −0.1 e). These results demonstrate that several LMCT and IL transitions are accessible at low energies for **1a**, which may explain the broad absorption band for this complex observed experimentally.

### Synthesis and Characterization of An^IV^(^Mes^PDP^Ph^)_2_ Complexes

Returning to our initial aim to isolate An(^Mes^PDP^Ph^)_2_ compounds, new synthetic protocols were developed starting from complexes **1a** and **1b**. No reaction is observed when 1 equiv of *in situ*-generated Li_2_^Mes^PDP^Ph^ in THF is added to complex **1a**, even under prolonged reaction periods at elevated temperatures. This is likely a result of the bulky nature of the (^Mes^PDP^Ph^)^2–^ ligand. To probe this hypothesis, we attempted the synthesis of the analogous bis-ligand complexes, featuring the much less bulky pyridine dipyrrolide ligands, (^Ph^PDP^Ph^)^2–^. These compounds can be synthesized at room temperature directly from the salt metathesis reaction of 2 equiv of *in situ*-prepared Li_2_^Ph^PDP^Ph^ and 1 equiv of AnCl_4_ (An = UCl_4_ or ThCl_4_(DME)_2_) in Et_2_O or toluene. (See Figures S7 and S8 for the characterization of these complexes by ^1^H NMR and Table S2 for crystallographic details.)

**Scheme 2:**
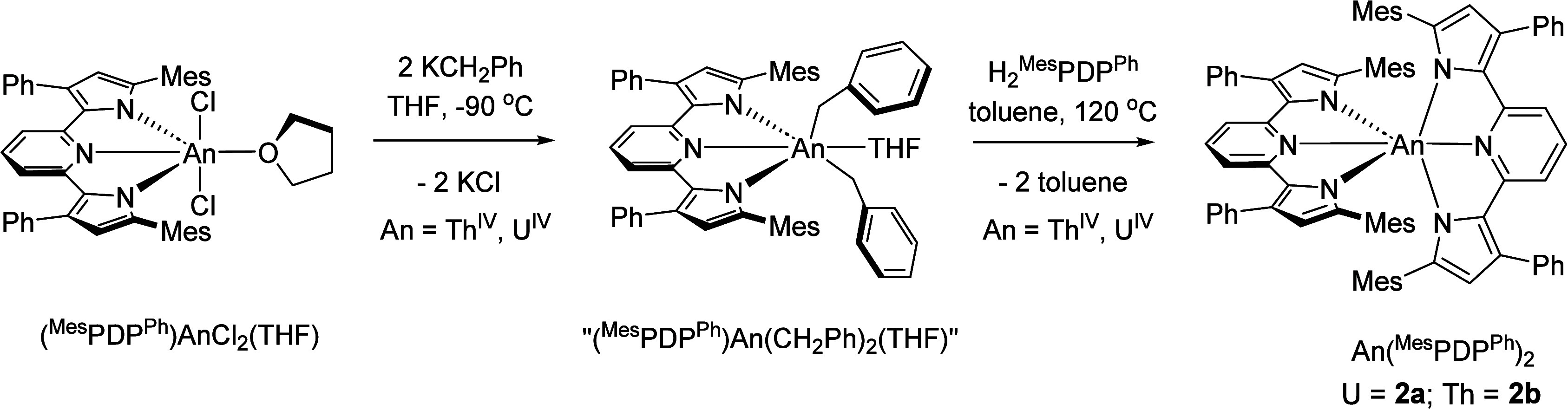
Synthesis of An(^Mes^PDP^Ph^)_2_ (An = U, **2a**; Th, **2b**)

Given the challenges noted in the attempted salt metathesis reactions for the coordination of a second pincer ligand, we proposed instead to alkylate the monoligated uranium(IV) compound, hypothesizing that the resultant uranium–carbon bonds would serve as an internal base to drive the formation of U(^Mes^PDP^Ph^)_2_. Indeed, evidence supporting the need to access an organometallic intermediary complex to isolate bis-PDP compounds was shown in a recent work by some of us; a cyclometalated Zr compound, (cyclo-^Mes^PDP^Ph^)ZrBn, was crucial to facilitate bis-ligand complex formation.^76^ The addition of 2 equiv of benzyl potassium (KCH_2_Ph) to (^Mes^PDP^Ph^)UCl_2_(THF) in THF at −80 °C resulted in an immediate color change from red to burgundy. Following a brief workup (see Experimental Section for details), the likely product of alkylation was resuspended in toluene and a second equivalent of H_2_^Mes^PDP^Ph^ was added; subsequently, a gradual color change to bright red-orange was observed. After heating at 120 °C for 32 h, purification of the crude reaction mixture afforded complex U(^Mes^PDP^Ph^)_2_ (**2a**) in good yield (69%; Scheme 2, see Experimental Section for additional details).

The formation of a new uranium-containing product was confirmed by ^1^H NMR spectroscopy. Nine paramagnetically shifted and broadened resonances ranging from +19 to −9 ppm were observed, consistent with the formation of the anticipated *D*_*2d*_-symmetric product (Figure S9). The ^1^H NMR spectrum of **2a** possesses a triplet resonance that integrated to 2, assigned to the 4-pyridyl protons of the PDP ligand at −3.35 ppm. A diagnostic singlet resonance attributed to the 4-pyrrolide hydrogens was present at 18.38 ppm, and a doublet appearing at −8.31 ppm represents the 3-pyridyl hydrogens. The highest-intensity peak in the spectrum is observed at 12.17 ppm, with the relative integration of 24 corresponding to the *ortho*-methyl protons of the mesityl group. A signal corresponding to the *para*-methyl protons of the PDP mesityl group is located at −2.83 ppm. Both resonances are shifted downfield in comparison to the ^1^H NMR spectrum of **1a**, indicating that the protons are significantly deshielded upon coordination of the second ligand equivalent to the paramagnetic actinide center.

**Figure 7:**
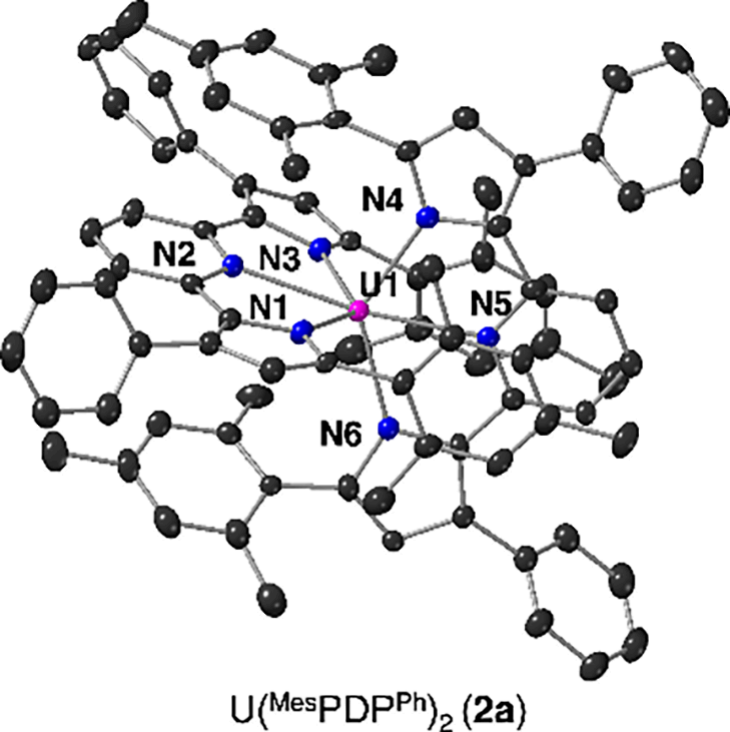
Molecular structure of U(^Mes^PDP^Ph^)_2_ (**2a**) shown with 30% probability ellipsoids. The molecular structure of Th(^Mes^PDP^Ph^)_2_ (**2b**) is analogous to **2a**; the corresponding image can be found in the Supporting Information (Figure S11). Hydrogen atoms and solvent molecules have been removed for clarity. Key: pink, U; blue, N; gray, C.

Single crystals of **2a** suitable for single crystal X-ray diffraction (SCXRD) were grown from a concentrated solution of the compound in neat diethyl ether at −30 °C. Refinement of the data confirmed formation of the anticipated six-coordinate, bis-ligand complex **2a** (Figure 7, Table 2). The coordination environment around uranium is best described as distorted octahedral with two meridionally bound N_3_-(^Mes^PDP^Ph^)^2–^ ligands. The dative interaction between the U–N_pyridine_ (N_pyridine_ = N2, N5) moieties results in bond lengths of 2.408(4) and 2.404(4) Å, further shortened in comparison to monoligated complex **1a** (U–N_pyridine_ = 2.474(4) Å). The U–N_pyrrolide_ (N_pyrrolide_ = N1, N3, N4, N6) bond distances (2.338(4) Å (average of N1 and N3), 2.313(4) Å (average of N4 and N6)) are elongated from the analogous bonds in **1a** (2.309(5) Å, 2.301(5) Å). This is likely a result of the steric congestion surrounding the uranium metal center upon coordination of two (^Mes^PDP^Ph^)^2–^ ligands. The N_pyrrolide_–M–N_pyrrolide_ bond angles for the pyridine dipyrrolide ligands in **2a** are 137.25(13) and 136.71(13)°; these values are slightly decreased from those reported for Zr(^Mes^PDP^Ph^)_2_ (N_pyrrolide_–Zr–N_pyrrolide_ = 143.54(8)°).^33^ We hypothesize that this is due to the large ionic radius of U(IV) (0.89 Å) in comparison to that of Zr(IV) (0.72 Å), which displaces the metal center from the optimal coordination pocket of the tridentate ligand.^77^ Consequently, a striking difference that results from this is the decrease in linearity of the N2–M–N5 bond angle in **2a** (N2–U–N5 = 173.31(12)°) as compared to Zr(^Mes^PDP^Ph^)_2_ (N2–Zr–N5 = 179.42(8)°).

**Table 2:** Pertinent Bond Distances and Angles for Complexes An(^Mes^PDP^Ph^)_2_ (An = U, **2a**; Th, **2b**); Distances and Angles for Zr(^Mes^PDP^Ph^)_2_^REF^ Included for Comparison

Complex	U(^Mes^PDP^Ph^)_2_ (**2a**)	Th(^Mes^PDP^Ph^)_2_ (**2b**)	Zr(^Mes^PDP^Ph^)_2_
M–N_pyridine_	2.408(4) Å	2.489(5) Å	2.262(2) Å
	2.404(4) Å	2.496(5) Å	2.262(2) Å
M–N_pyrrolide_	2.328(4), 2.347(4) Å	2.381(4), 2.409(4) Å	2.170(2), 2.166(2) Å
	2.297(4), 2.329(3) Å	2.348(4), 2.384(4) Å	2.170(2), 2.165(2) Å
N2–M–N5	173.31(12)°	171.79(14)°	179.42(8)°

With complex **2a** isolated, we targeted the preparation of the isostructural thorium analogue, Th(^Mes^PDP^Ph^)_2_ (**2b**), following a procedure similar to that invoked in the successful formation of the uranium derivative. The addition of 2 equiv of KCH_2_Ph to (^Mes^PDP^Ph^)ThCl_2_(THF) in THF at −80 °C resulted in an immediate color change from yellow to bright red. Upon workup, the benzyl-substituted complex ^Mes^PDP^Ph^Th(CH_2_Ph)_2_(THF) was redissolved in toluene and 1 equiv of H_2_^Mes^PDP^Ph^ suspended in toluene was added, resulting in a color change to orange. After heating to 120 °C for 32 h, workup of the solution afforded **2b** in good yield (77%; Scheme 2). Characterization of the product by ^1^H NMR spectroscopy revealed the expected number of resonances with relative integrations for a *D*_*2d*_-symmetric product in solution (Figure S10). Diagnostic signals observed in the ^1^H NMR spectrum of **2b** include an apparent triplet resonance at 6.53 ppm that overlaps with an aryl-proton peak, assigned to the 4-pyridyl protons of the PDP ligand backbone. A singlet resonance corresponding to the 4-pyrrolide hydrogen atoms appears at 6.04 ppm, whereas the *ortho*-mesityl and *para*-mesityl methyl protons are observed as singlet resonances located upfield at 2.15 and 1.94 ppm, respectively. ^13^C NMR of **2b** was also obtained and is consistent with the anticipated spectrum of the product (Figure S12).

Yellow crystals of **2b** suitable for analysis via SCXRD were obtained from a concentrated diethyl ether solution of the compound at −30 °C. Refinement of the data confirmed the identity of complex **2b** as the desired pseudo-octahedral, six-coordinate species Th(^Mes^PDP^Ph^)_2_ (Figure 7, Table 2). The bis-ligated thorium complex displays average Th–N_pyrrolide_ bond lengths of 2.395(4) and 2.366(4) Å, similar to other reported Th–N_pyrrolide_ bond distances.^73^ The N_pyrrolide_–M–N_pyrrolide_ bond angles for the pincer ligands in **2b** are 133.75(15) and 132.32(15)°, slightly decreased from those in **2a** and Zr(^Mes^PDP^Ph^)_2_ due to the increased size of the thorium ion (Th(IV) = 0.94 Å). Bond lengths for Th–N_pyridine_ in Th(^Mes^PDP^Ph^)_2_ are significantly shortened at 2.489(5) and 2.496(5) Å compared to a Th(IV) porphyrin complex that reports a Th–N_pyridine_ bond length of 2.614 Å;^21^ this is consistent with the overall truncation in An–N bond distances that is observed in this series of An–PDP complexes.

**Figure 8:**
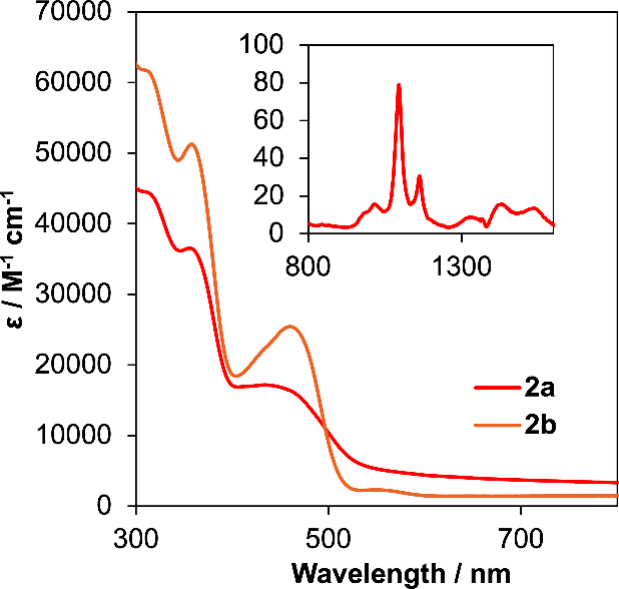
Electronic absorption spectra for U(^Mes^PDP^Ph^)_2_ (**2a**) and Th(^Mes^PDP^Ph^)_2_ (**2b**) collected at room temperature in toluene. The inset shows the near-infrared region of the electronic absorption spectrum of **2a** to highlight f–f transitions of the U(IV) center.

Analysis of the optical properties of the bis-ligated complexes, **2a** and **2b**, was performed by electronic absorption spectroscopy (Figure 8). Complexes **2a** and **2b** exhibit spectra similar to those of the monoligated derivatives. The absorption profile of Th(^Mes^PDP^Ph^)_2_ displays two intense bands located at 316 nm (ε = 61,077 M^–1^ cm^–1^) and 360 nm (ε = 51,105 M^–1^ cm^–1^) as well as a lower-energy feature at 462 nm (ε = 25,397 M^–1^ cm^–1^). In comparison to complex **1b**, **2b** possesses higher molar extinction coefficients for all three transitions observed in the electronic absorption spectrum. This is consistent with the addition of a second equivalent of ligand to the thorium center, as all transitions are proposed to be ligand-based. The electronic absorption spectrum of **2a** likewise features two intense bands at 320 nm (ε = 43,317 M^–1^ cm^–1–^) and 358 nm (ε = 36,466 M^–1^ cm^–1–^), along with a weaker absorption noted at 450 nm (ε = 16,839 M^–1^ cm^–1^). As observed in the case of **1a**, this absorption feature extends far into the visible region of the spectrum. Analysis of the near-infrared region of the spectrum of **2a** also reveals weak and sharp f–f transitions, consistent with the retention of a +IV oxidation state of uranium upon addition of the second PDP ligand to the metal center. Complex **2a** also shows higher molar extinction coefficients in comparison to **1a**; overall, the coordination of a second (^Mes^PDP^Ph^)^2–^ ligand to both actinide centers increases the probability of the allowed transitions to occur in the electronic absorption spectra of these compounds.

**Figure 9:**
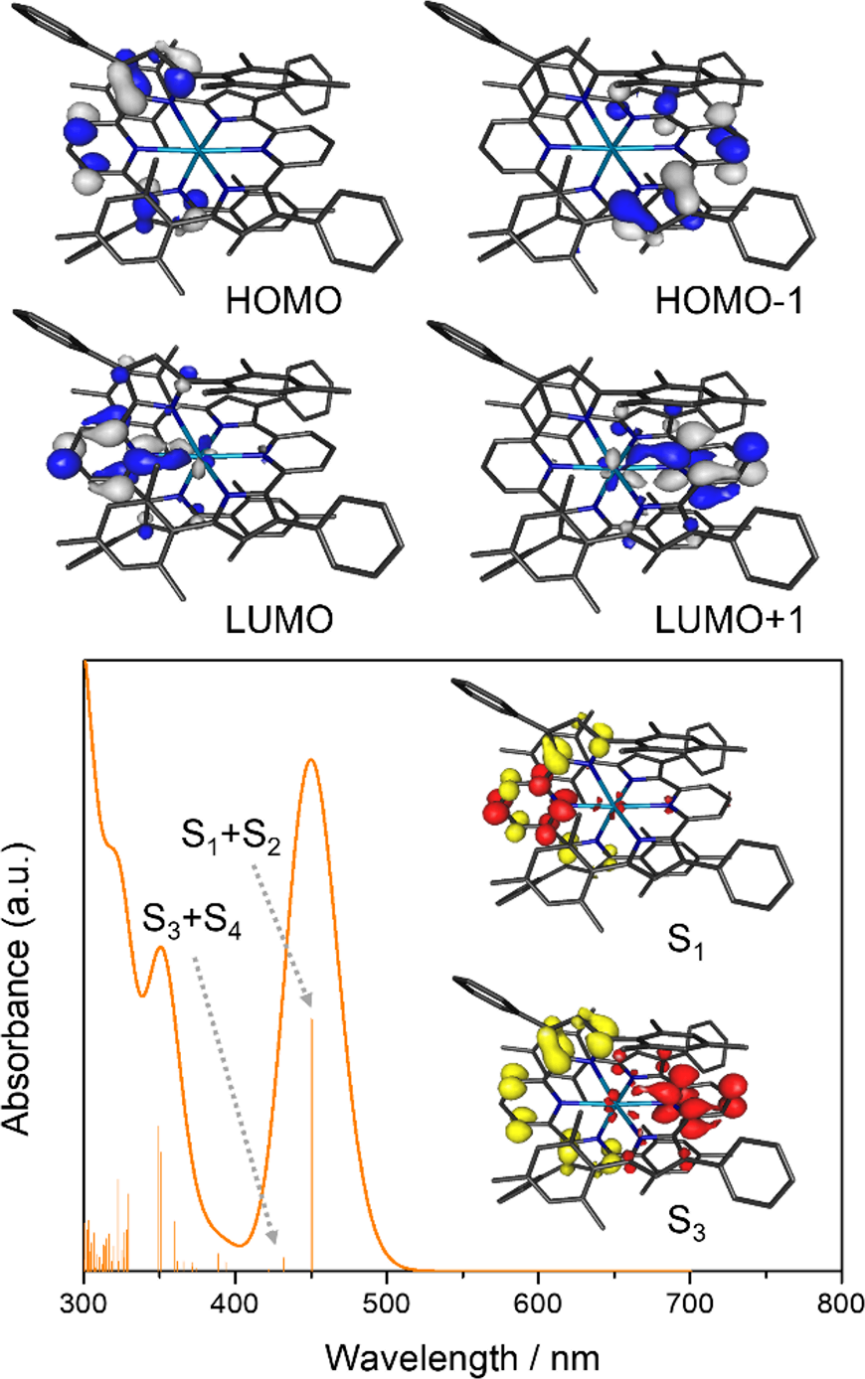
Top: Depictions of the degenerate sets of HOMO/HOMO–1 and LUMO/LUMO+1 of Th(^Mes^PDP^Ph^)_2_ obtained by DFT calculations. Bottom: TD-DFT calculated absorption spectrum (fwhm = 2000 cm^–1^) with individual transitions indicated by the stick plot. The insets show the difference densities for the lowest-energy excited states (red = increased electron density, yellow = reduced electron density). The calculated S_2_ and S_4_ states are degenerate with respect to S_1_ and S_3_, respectively, and the corresponding difference densities can be obtained by a *C*_2_ operation interconverting the two ^Mes^PDP^Ph^ ligands.

Computational analysis of the electronic structures and electronic transitions of U(^Mes^PDP^Ph^)_2_ and Th(^Mes^PDP^Ph^)_2_ was performed using the same computational approaches as described for monoligand complexes **1a** and **1b**. Like that noted in the case of the analysis of complex **1b**, electronic structure analysis for diamagnetic thorium complex **2b** proved to be straightforward and confirmed the presence of a Th(IV) center (Figure 9). All filled frontier molecular orbitals are exclusively ligand-centered with negligible contributions from the metal ion (<3%). The HOMO and HOMO–1 are nearly degenerate and exhibit major contributions from the pyrrolide π systems. Similar to the structurally related group 4 complexes Zr(^Mes^PDP^Ph^)_2_ and Hf(^Mes^PDP^Ph^)_2_, the LUMO and LUMO+1 form a degenerate orbital set (e) under idealized *D_2d_* symmetry. In contrast to its transition metal congeners, however, the LUMO/LUMO+1 of **2b** exhibit only minor contributions from the Th center (10%) and contain dominant contributions from the pyridine rings of the two PDP ligands more akin to the main group compounds E(^Me^PDP^Ph^)_2_ (E = Si, Ge, Sn).

TD-DFT calculations for **2b** at the B3LYP/DKH level of theory predict a lowest-energy absorption band at 451 nm that is composed of two sets of degenerate transitions from the HOMO (a_2_) to the LUMO/LUMO+1 (e, S_1_ and S_2_) and from the HOMO–1 (b_2_) to the LUMO/LUMO+1 (S_3_ and S_4_) (Figure 9). Mulliken population analysis for the ground-state and the corresponding excited-state electron densities revealed only minor charge migration to the Th center (Δ*q*_1,2_ = −0.08 e; Δ*q*_3,4_ = −0.10 e), corroborating the intraligand (^1^IL) and singlet ligand-to-ligand charge transfer (^1^LLCT) character of the excited states. This is consistent with the orbital compositions of the donor and acceptor orbitals established for the ground-state electronic structure. A second absorption band computed at 350 nm can also be attributed to several higher-energy ^1^IL and ^1^LLCT transitions with minimal charge migration to the metal (Δ*q* < −0.12 e).

**Figure 10:**
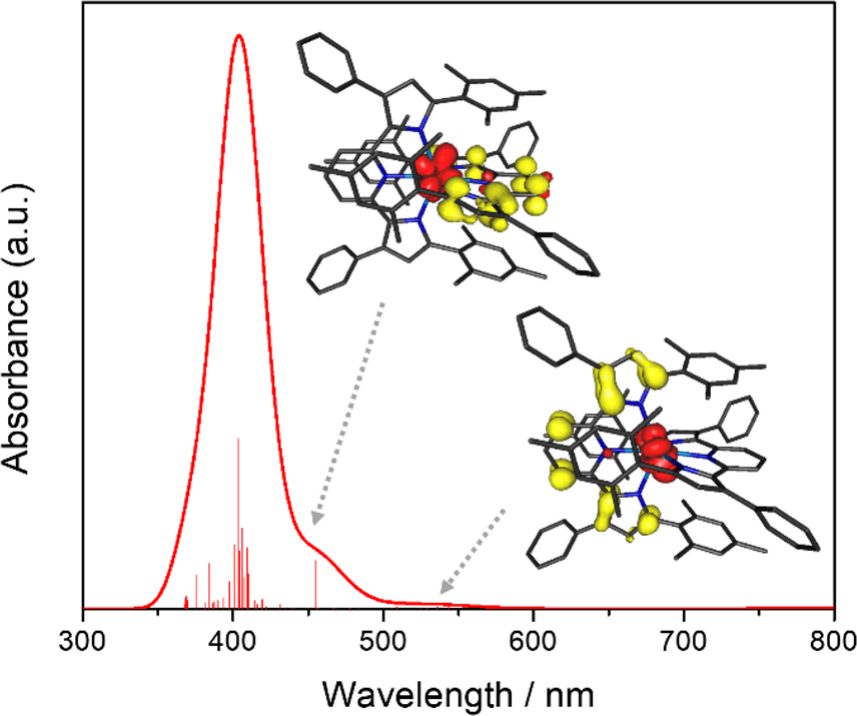
TD-DFT calculated absorption spectrum for U(^Mes^PDP^Ph^)_2_ (fwhm = 2000 cm^–1^) including only the first 50 roots with individual transitions indicated by the stick plot. The insets show the difference densities for the two lowest-energy charge transfer excited states (red = increased electron density, yellow = reduced electron density), highlighting their ^1^LMCT character.

Calculations for uranium analog **2a** assuming a paramagnetic triplet ground state support a +IV oxidation state with an f^2^ electron configuration for the metal center, reflected in a spin density of 2.09 obtained by Mulliken population analysis. The HOMO and HOMO–1 for both the α- and β-orbital manifolds are degenerate under idealized *D*_*2d*_ symmetry and exhibit exclusively ligand character much like the equivalent HOMO/HOMO–1 set for **2b**. While the degenerate LUMO and LUMO+1 for the β-manifold exhibits only minor contributions from the uranium center, the corresponding orbitals in the α-set show substantial f-orbital contributions with 89.7 and 81.1% metal character. Consistent with these assignments, TD-DFT calculations predict several low-energy ^3^LMCT transitions between 550 and 350 nm, resulting in significant charge and spin density migration to the uranium center (Figure 10).

### Luminescence Studies

We next conducted a preliminary investigation of the photophysical properties of our isostructural PDP An(IV) compounds. As described at the outset of this report, considering the remarkable photoluminescence properties of Sn^IV^(^Me^PDP^Ph^)_2_ (5s^0^5p^0^),^36^ Zr^IV^(^Mes^PDP^Ph^)_2_ (4d^0^),^33^ and Hf^IV^(^Mes^PDP^Ph^)_2_ (5d^0^),^76^ thorium complex **2b** represents a logical and convenient expansion into the f-block elements. Direct comparison of **2b** (5f^0^) to its uranium analogue **2a** (5f^2^) provides a route to probe the influence of energetically accessible 5f orbitals and f–f excited states on luminescence of these complexes. Notably, readily accessible d–d excited states have been well-established to facilitate a rapid nonradiative decay in transition metal complexes.^78^ Finally, comparing mono- and bis-ligated PDP complexes may illustrate whether two (^Mes^PDP^Ph^)^2–^ ligands are required to achieve interesting optical properties in metal complexes of this type.

**Figure 11:**
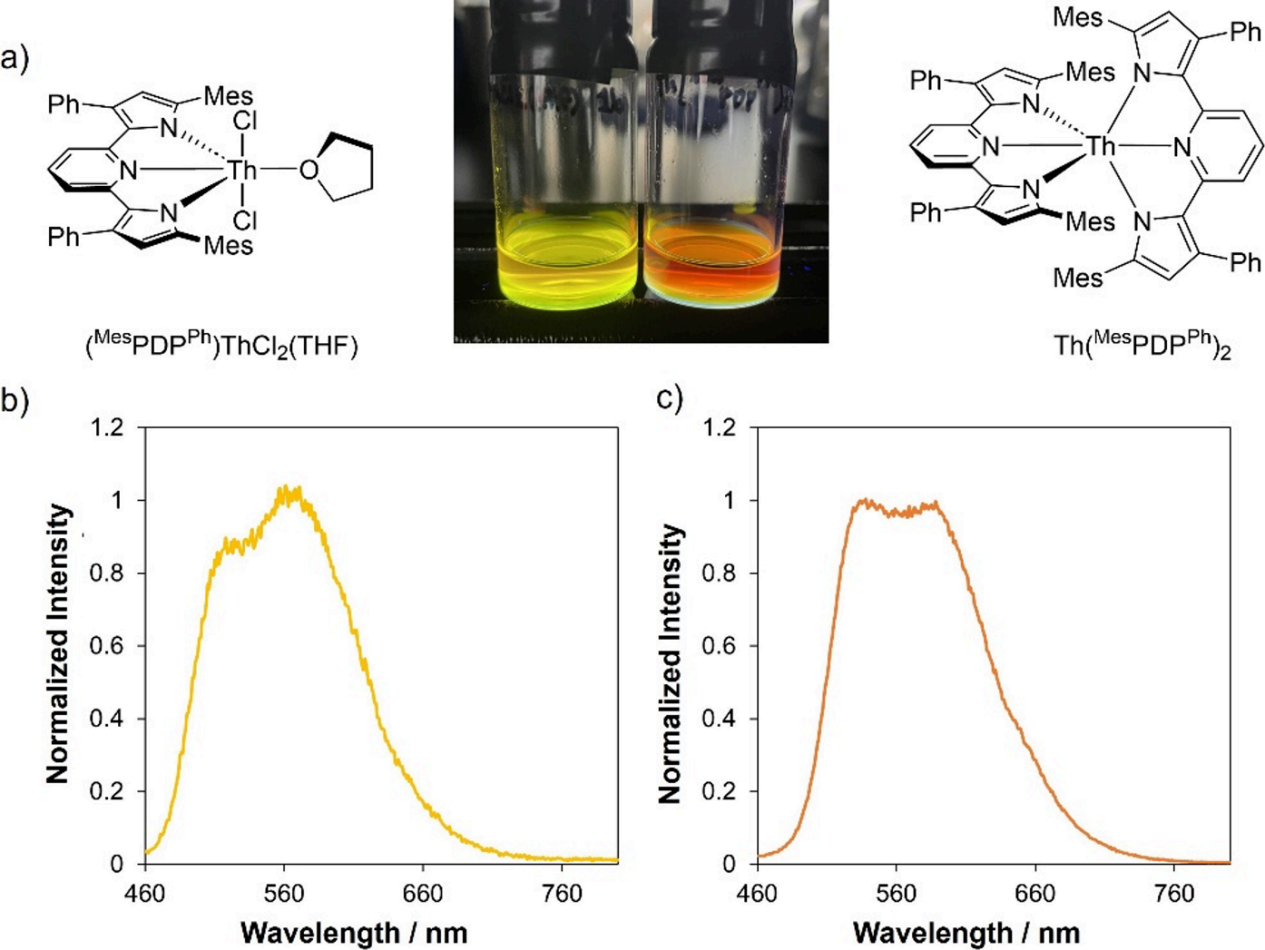
(a) Molecular structures of complexes **1b** and **2b**; the photograph includes an image of samples of **1b** (left) and **2b** (right) irradiated in toluene under 365 nm light. (b) Emission recorded at room temperature for **1b** in toluene. (c) Emission recorded at room temperature for **2b** in toluene.

We begin our discussion with Th(^Mes^PDP^Ph^)_2_, due to its relation to other M(^Mes^PDP^Ph^)_2_ (M = Zr, Hf, Sn) complexes that have been reported previously. Excitation of Th(^Mes^PDP^Ph^)_2_ in an anhydrous toluene solution at room temperature with UV or visible light below 500 nm resulted in strong photoluminescence (Figure 11). The spectral profile features two broad, overlapping bands, with maxima at 548 and 586 nm. A photoluminescence quantum yield of 42% was determined for **2b** at room temperature by the comparative method (Φ_PL_ = 0.42 ± 0.01; Figure S13). This value indicates a high quantum efficiency for complex **2b** and is substantially larger than values reported previously for Th(IV) complexes (e.g., [Li(THF)_2_][Th = NAr^3,5-CF3^(TriNOx)]; QY = 2.5%).^79^ The photoluminescence decay for Th(^Mes^PDP^Ph^)_2_ revealed a lifetime of 304 μs, which we hypothesize to be due to the decay of a long-lived triplet excited state (Figure S14).^85^

It is instructive to compare the photoluminescence properties of **2b** with those of its transition metal and main group analogs Zr(^Mes^PDP^Ph^)_2_, Hf(^Mes^PDP^Ph^)_2_, and Sn(^Me^PDP^Ph^)_2_ (Table 3). All three reference compounds exhibit long-lived photoluminescence with lifetimes of hundreds of microseconds to milliseconds and high quantum yields resulting from thermally activated delayed fluorescence (TADF) at room temperature. The similar values for lifetime and quantum yield observed in the present study suggest that a similar photoluminescence mechanism may be at play for Th(^Mes^PDP^Ph^)_2_. However, there are minor differences in the photophysical data that hint at subtle changes in the electronic structure among the four compounds. These may be a consequence of the difference in valence orbitals for main group, d-block, and f-block elements. Due to the very limited contributions of the Sn p orbitals to the frontier molecular orbitals of Sn(^Me^PDP^Ph^)_2_, the lowest-energy excited state is predominantly ligand-centered. Consequently, intersystem crossing (ISC) to the triplet state is relatively slow, resulting in the observation of competing prompt and delayed fluorescence at room temperature (k_ISC_ ≈ k_PF_). In contrast, significant d-orbital contributions to the LUMOs of Zr(^Mes^PDP^Ph^)_2_ and Hf(^Mes^PDP^Ph^)_2_ introduce LMCT character into the excited state, facilitating rapid ISC (k_ISC_ ≫ k_PF_) and exclusively TADF under ambient conditions. Th(^Mes^PDP^Ph^)_2_ presents an interesting case because it maximizes heavy-atom effects compared to the remaining three compounds but shows metal contributions that are in-between its main group and transition metal analogs. Highlighting its unusual position within the series, the room-temperature emission spectrum of Th(^Mes^PDP^Ph^)_2_ exhibits two emission maxima, while the remaining group 4 and 14 compounds show only one broad absorption feature. More detailed studies of the photophysics of Th(^Mes^PDP^Ph^)_2_ are required to firmly establish the nature of the electronic transitions responsible for emission but are beyond the scope of this initial report.

**Table 3:** Comparison of Optical Properties of M(^R^PDP^Ph^)_2_ Complexes (M = Th, Zr, Hf; R = Mesityl) (M = Sn; R = Methyl)

**Complex**	**Th(^Mes^PDP^Ph^)_2_**	**Zr(^Mes^PDP^Ph^)_2_**	**Hf(^Mes^PDP^Ph^)_2_**	**Sn(^Me^PDP^Ph^)_2_**
**λ**_**abs**_ **(nm)**	462	525	507	464
**λ**_**em**_ **(nm)**	548, 586	581	558	512
**Φ**_**PL**_	42%	45%	41%	32%
**τ (ms)**	0.304	0.350	0.450	2.0
**Reference**	This work	33	76	36

Next, we investigated the luminescence of the mono-PDP thorium complex to determine the effect of the number of coordinated PDP ligands on the resulting photophysical properties. Excitation of the lowest-energy absorption band in (^Mes^PDP^Ph^)ThCl_2_(THF) in anhydrous toluene at room temperature resulted in strong photoluminescence (Figure 11). The emission profile is similar to that of **2b**, featuring two broad, superimposed bands with maxima at 532 and 568 nm and an energy separation of 1,241 cm^–1^. Coordination of only one (^Mes^PDP^Ph^)^2–^ ligand in **1b** causes a blue shift in the emission profile of the complex in comparison to that of **2b** and a slight increase in emission intensity at room temperature. Emission decay kinetics of **1b** display similar behavior to that of **2b**, with a lifetime of τ = 256 μs. (Figure S14). This observation suggests that the number of coordinated PDP ligands does not have a drastic effect on the decay pathways of these systems. In addition, the quantum yield determined for **1b** by the comparative method is comparable to that of **2b** at 45% (Φ_e_ = 0.45 ± 0.02) (Figure S13).

It is important to note that in contrast to the thorium(IV) congeners, excitation of (^Mes^PDP^Ph^)UCl_2_(THF) and U(^Mes^PDP^Ph^)_2_ in anhydrous toluene solutions at room temperature with UV or visible light below 600 nm resulted in no observable emission for both complexes. We believe that the emission decay pathway in the case of the uranium complexes is proceeding through the 5f orbital manifold due to the low-energy ^3^LMCT transitions present in **1a** and **2a**, which have been affirmed by TD-DFT calculations.

## Conclusions

Here, we report the synthesis and characterization of four actinide complexes of the pyridine dipyrrolide ligand class, (^Mes^PDP^Ph^)AnCl_2_(THF) and An(^Mes^PDP^Ph^)_2_ (An = U, Th). The title compounds have been characterized thoroughly through solid- and solution-state methods. The electronic absorption spectra of all complexes are dominated by two high-energy charge transfer bands with predominantly π–π* character, similar to previously reported transition metal and metalloid adducts of the PDP ligand. In the case of the uranium derivatives, evidence for LMCT is observed in the electronic absorption spectrum, characterized by low-energy transitions ranging from 500 to 600 nm, which has also been corroborated with TD-DFT calculations. In contrast, the thorium derivatives exhibit exclusively ILCT and LLCT transitions, consistent with the very negative reduction potential and high energy of the 5f orbitals of Th(IV) ions. Additionally, we report strong room-temperature photoluminescence for the thorium derivatives, with high quantum efficiencies ranging from 40 to 45%. More detailed temperature-dependent and time-resolved studies are required to elucidate the excited-state dynamics of ^Mes^PDP^Ph^ThCl_2_(THF) and Th(^Mes^PDP^Ph^)_2_, which is beyond the scope of this initial report.

## Supporting Information Available

Additional experimental procedures, spectroscopic and crystallographic data, and computational details (PDF)
