# Supplementary material for: Synthesis and Characterization of Isostructural Th(IV) and U(IV) Pyridine Dipyrrolide Complexes
Source: Inorg Chem. 2024 Feb 20;63(21):9610–23. doi: 10.1021/acs.inorgchem.3c04391 (PMC11134498; doi:10.1021/acs.inorgchem.3c04391)
Supplement: Supplementary file 1 — ic3c04391_si_001.pdf [file ic3c04391_si_001.pdf]

# Electronic Supporting Information

# Synthesis and Characterization of Isostructural Th(IV) and U(IV) Pyridine Dipyrrolide Complexes

Leyla R. Valerio[1], Brett M. Hakey[1], Dylan C. Leary[2], Erin Stockdale[1], William W. Brennessel[1], Carsten Milsmann[2*], Ellen M. Matson[1*]

[1] *Department of Chemistry, University of Rochester, Rochester NY 14627 USA*

[2] *C. Eugene Bennett Department of Chemistry, West Virginia University, Morgantown, West Virginia 26506, USA*

**Corresponding Author Contact Information:**

Carsten Milsmann: camilsmann@mix.wvu.edu

Ellen M. Matson: matson@chem.rochester.edu

**Supporting Information Table of Contents**

**Synthesis of U($^{Ph}$PDP$^{Ph}$)$_2$** ……………………………………………………………………………… 3

**Synthesis of Th($^{Ph}$PDP$^{Ph}$)$_2$** ……………………………………………………………………………...4

**Table S1**. Crystallographic parameters for M($^{Ph}$PDP$^{Ph}$)$_2$ (M =U,Th)……………………………………….5

**Table S2**. Crystallographic parameters for $^{Mes}$PDP$^{Ph}$MCl$_2$(THF) (M =U,Th)……………………………6

**Table S3**. Crystallographic parameters for M($^{Mes}$PDP$^{Ph}$)$_2$ (M =U,Th)……………………………………7

**Figure S1.** $^1$H NMR spectrum of attempt of one-pot synthesis of U($^{Mes}$PDP$^{Ph}$)$_2$……………………………..8

**Figure S2.** $^1$H NMR spectrum of $^{Mes}$PDP$^{Ph}$UCl$_2$(THF) (**1a**)…………………………………………………..9

**Figure S3.** $^1$H NMR spectrum of $^{Mes}$PDP$^{Ph}$ThCl$_2$(THF) (**1b**)…………………………………………..…10

**Figure S4.** SCXRD structure of $^{Mes}$PDP$^{Ph}$ThCl$_2$(THF) (**1b**)……………………………………………..11

**Figure S5.** $^{13}$C NMR spectrum of $^{Mes}$PDP$^{Ph}$ThCl$_2$(THF) (**1b**)…………………………………………....12

**Figure S6.** UV-Vis spectrum of H$_2$$^{Mes}$PDP$^{Ph}$………………………………………………………………....13

**Figure S7.** $^1$H NMR spectrum of U($^{Ph}$PDP$^{Ph}$)$_2$…………………………………………………………….14

**Figure S8.** $^1$H NMR spectrum of Th($^{Ph}$PDP$^{Ph}$)$_2$……………………………………………………………15

**Figure S9.** $^1$H NMR spectrum of U($^{Mes}$PDP$^{Ph}$)$_2$ (**2a**)……………………………………………………16

**Figure S10.** $^1$H NMR spectrum of Th($^{Mes}$PDP$^{Ph}$)$_2$ (**2b**)…………………………………………………..17

**Figure S11.** SCXRD structure of Th($^{Mes}$PDP$^{Ph}$)$_2$ (**2b**)…………………………………………………….18

**Figure S12.** $^{13}$C NMR spectrum of Th($^{Mes}$PDP$^{Ph}$)$_2$ (**2b**)…………………………………………………..19

**Figure S13.** Quantum yield determination via comparative method………………………………..20

**Figure S14.** Excited State Lifetime Decay Fits of **2b** and **1b**……………………………………………21

**Calculation Input Files**…………………………………………………………………………………..22

**Synthesis of U($^{Ph}$PDP$^{Ph}$)$_2$**

*Caution! Depleted uranium (primary isotope $^{238}$U) is a weak α-emitter (4.197 MeV) with a half-life of 4.47 x $10^9$ years; manipulations and reactions should be carried out in monitored fume hoods or in an inert atmosphere drybox in a radiation laboratory equipped with α- and β counting equipment.*

$H_2$$^{Ph}$PDP$^{Ph}$ and $UCl_4$ were synthesized following the literature reported procedures.[1,2]

In the glovebox, a 20 mL scintillation vial equipped with a magnetic stirrer was loaded with 100 mg (0.195 mmol) of H2$^{Ph}$PDP$^{Ph}$ and 3 mL of diethyl ether, affording a yellow suspension. In a separate vial, 66 mg (0.395 mmol, 2.03 eq.) of LiHMDS was dissolved in 3 mL of diethyl ether. The LiHMDS solution was added to the suspension of H2$^{Ph}$PDP$^{Ph}$ with vigorous stirring, inducing an immediate color change to a brilliant luminescent yellow. The resulting suspension was stirred for 2 hrs. In a separate vial, 44 mg of $UCl_4$ (0.116 mmol, 0.59 eq.) was suspended in 3 mL of diethyl ether and added to the suspension of $Li_2$$^{Ph}$PDP$^{Ph}$, inducing an immediate color change to dark-red. The mixture was stirred for 16 hrs, at which time it was filtered over a 1" pad of celite supported by a glass microfiber plug. The red powder was washed three times with 1 mL aliquots of pentane and then extracted into DCM (10 mL) and collected in a 20 mL scintillation vial. Removal of volatiles in vacuo, followed by trituration with pentane afforded a red powder identified as the title compound. Yield: 86 mg, 0.068 mmol, 70%. $^1$H NMR (400 MHz, benzene-d6) δ 25.36 (s, 2H, pyrrole*H*), 9.51 (d, J = 7.6 Hz, 4H, ortho-Ph*H*), 7.89 (t, J = 7.6 Hz, 4H, meta-Ph*H*), 7.42 (t, J = 7.6 Hz, 2H, para-Ph*H*), 1.43 (t, J = 6.7 Hz, 2H, para-Ph*H*), 0.13 (s, 4H, Ar*H*), -0.63 (t, J = 7.4 Hz, 1H, 4-py*H*), -3.74 (d, J = 7.5 Hz, 2H, 3-py*H*), -7.28 (s, 4H, Ar*H*). Dark red blocks of U($^{Ph}$PDP$^{Ph}$)$_2$ suitable for X-ray crystallography were grown from a mixture of toluene and diethyl ether at -30°C. Anal. Calcd for $UOC_{85}H_{68}N_6$: C, 71.52; H, 4.80; N, 5.89. Found: C, 71.30; H, 4.55; N, 5.56.

**Synthesis of Th($^{Ph}$PDP$^{Ph}$)$_2$**

*Caution! $^{232}$Th is a weak α-emitter (4.082 MeV) with a half-life of 1.41 x $10^{10}$ years; manipulations and reactions should be carried out in monitored fume hoods or in an inert atmosphere drybox in a radiation laboratory equipped with α- and β counting equipment.*

$ThCl_4(DME)_2$ was synthesized following a reported procedure.[3]

In the glovebox, a 20 mL scintillation vial equipped with a magnetic stirrer was loaded with 100 mg (0.195 mmol) of $H_2{}^{Ph}PDP^{Ph}$ and 3 mL of toluene, affording a yellow solution. In a separate vial, 66 mg (0.395 mmol, 2.03 eq.) of LiHMDS was dissolved in 3 mL of toluene. The LiHMDS solution was added to the suspension of $H_2{}^{Ph}PDP^{Ph}$ with vigorous stirring, inducing an immediate color change to a luminescent dark orange. The resulting suspension was stirred for 2 hrs. In a separate vial, 54 mg of $ThCl_4(DME)_2$ (0.097 mmol, 0.50 eq.) was suspended in 3 mL of toluene and added to the suspension of $Li_2{}^{Ph}PDP^{Ph}$, inducing an immediate color change to bright orange. The mixture was stirred for 16 hrs, at which time it was filtered over a 1" pad of celite supported by a glass microfiber plug and collected in a 20 mL scintillation vial. Removal of volatiles in vacuo, followed by trituration with pentane afforded an orange powder identified as the title compound. $^1$H NMR (400 MHz, benzene-$d_6$) δ 7.70 (d, 2H), 7.60 (d, 4H), 7.34 (t, 4H), 7.23 (m, 4H), 7.05 (m, 2H), 6.92 (m, 4H), 6.68 (m, 2H), 6.47 (t, 1H), 6.37 (s, 2H). Anal. Calcd. for $C_{74}H_{48}N_6Th$ (mol. wt. 1253.278 g/mol): C, 73.26%; H, 4.89%; N, 5.7%. Found: C, 73.634%; H, 5.188%; N, 6.191%.

**References:**


1) Y. Zhang, D. C. Leary, A. M. Belldina, J. L. Petersen, C. Milsmann, *Inorg. Chem.,* 2020, **59**, 20, 14716–14730.

2) J. L. Kiplinger, D. E. Morris, B. L. Scott, C. J. Burns, *Organometallics* 2002, **21**, 5978–5982.

3) T. Cantat, B. L. Scott, J. L. Kiplinger, *Chem. Commun.,* 2010, **46**, 919-921.

**Table S1.** Crystallographic parameters for molecular structures of M($^{Ph}$PDP$^{Ph}$)$_2$ (M = U, Th).

| Compound | **U($^{Ph}$PDP$^{Ph}$)$_2$** | **Th($^{Ph}$PDP$^{Ph}$)$_2$** |
|---|---|---|
| Empirical Formula | $C_{93.5}$ $H_{75}$ $N_6$ $O_{0.5}$ U | $C_{91.04}$ $H_{73.60}$ $N_6$ $O_{0.76}$ Th |
| Formula Weight | 1528.62 | 1495.84 |
| Temperature / K | 100.00(10) | 100.00(10) |
| Wavelength / Å | 0.71073 | 1.54184 |
| Crystal Group | Monoclinic | Monoclinic |
| Space Group | $P2_{1/c}$ | $P2_{1/c}$ |
| Unit Cell Dimensions | a = 17.0198(4) Å<br>b = 15.6860(4) Å<br>c = 26.8680(7) Å<br>α = 90°<br>β = 96.348(2)°<br>γ = 90° | a = 17.12499(9) Å<br>b = 15.67015(9) Å<br>c = 27.00635(14) Å<br>α = 90°<br>β = 96.3084(5)°<br>γ = 90° |
| Volume / Å$^3$ | 7129.0(3) | 7203.30(7) |
| Z | 4 | 4 |
| Reflections Collected | 134273 | 26040 |
| Independent Reflections | 23680 | 26040 |
| Completeness (theta) | 99.9% (29.575°) | 99.8% (74.504°) |
| Goodness-of-fit on F$^2$ | 1.031 | 1.068 |
| Final R Indices [I>2sigma(I)] | *R*1 = 0.0339 | *R*1 = 0.0447 |
| Largest diff. peak and hole | 1.116 and -0.724 e.Å$^{-3}$ | 2.255 and -1.548 e.Å$^{-3}$ |

**Table S2.** Crystallographic parameters for molecular structures of **1a** and **1b**.

| Compound | $^{Mes}PDP^{Ph}UCl_2(THF)$ **1a** | $^{Mes}PDP^{Ph}ThCl_2(THF)$ **1b** |
|---|---|---|
| Empirical Formula | $C_{57.50}$ $H_{57}$ $Cl_2$ $N_3$ O U | $C_{57.50}$ $H_{57}$ $Cl_2$ $N_3$ O Th |
| Formula Weight | 1114.99 | 1109.00 |
| Temperature / K | 100.00(10) | 100.00(10) |
| Wavelength / Å | 1.54184 | 1.54184 |
| Crystal Group | Orthorhombic | Orthorhombic |
| Space Group | $P_{bcn}$ | $P_{bcn}$ |
| Unit Cell Dimensions | a = 31.0070(2) Å<br>b = 19.80190(12) Å<br>c = 15.78845(12) Å<br>α = 90°<br>β = 90°<br>γ = 90° | a = 30.9427(3) Å<br>b = 19.89950(10) Å<br>c = 15.85760(10) Å<br>α = 90°<br>β = 90°<br>γ = 90° |
| Volume / Å$^3$ | 9694.07(11) | 9764.23(12 |
| Z | 8 | 8 |
| Reflections Collected | 88138 | 87829 |
| Independent Reflections | 10451 | 10506 |
| Completeness (theta) | 99.9% (74.504°) | 99.99% (74.504°) |
| Goodness-of-fit on F$^2$ | 1.125 | 1.112 |
| Final R Indices [I>2sigma(I)] | $R1$ = 0.0417 | $R1$ = 0.0330 |
| Largest diff. peak and hole | 2.275 and -1.757 e.Å$^{-3}$ | 1.456 and -1.526 e.Å$^{-3}$ |

**Table S3.** Crystallographic parameters for molecular structures of **2a** and **2b**.

| Compound | U($^{Mes}PDP^{Ph}$)$_2$ **2a** | Th($^{Mes}PDP^{Ph}$)$_2$ **2b** |
|---|---|---|
| Empirical Formula | $C_{87}$ $H_{76.50}$ $N_6$ $O_{0.25}$ U | $C_{87}$ $H_{76.50}$ $N_6$ $O_{0.25}$ Th |
| Formula Weight | 1448.07 | 1442.08 |
| Temperature / K | 100.00(10) | 100.01(10) |
| Wavelength / Å | 1.54184 | 1.54184 |
| Crystal Group | Triclinic | Triclinic |
| Space Group | $P_{-1}$ | $P_{-1}$ |
| Unit Cell Dimensions | a = 16.25930(17) Å<br>b = 17.7247(2) Å<br>c = 26.1875(2) Å<br>α = 108.2640(9)°<br>β = 95.4111(8)°<br>γ = 106.1017(10)° | a = 16.3934(3) Å<br>b = 17.7407(3) Å<br>c = 26.0367(3) Å<br>α = 108.3900(10)°<br>β = 95.6750(10)°<br>γ = 105.9660(10)° |
| Volume / Å$^3$ | 6748.47(13) | 6763.80(19) |
| Z | 4 | 4 |
| Reflections Collected | 112008 | 108371 |
| Independent Reflections | 28686 | 28683 |
| Completeness (theta) | 99.8% (67.684°) | 99.8% (67.684°) |
| Goodness-of-fit on $F^2$ | 1.047 | 1.097 |
| Final R Indices [I>2sigma(I)] | $R1$ = 0.0453 | $R1$ = 0.0416 |
| Largest diff. peak and hole | 1.445 and -2.207 e.Å$^{-3}$ | 2.255 and -1.548 e.Å$^{-3}$ |

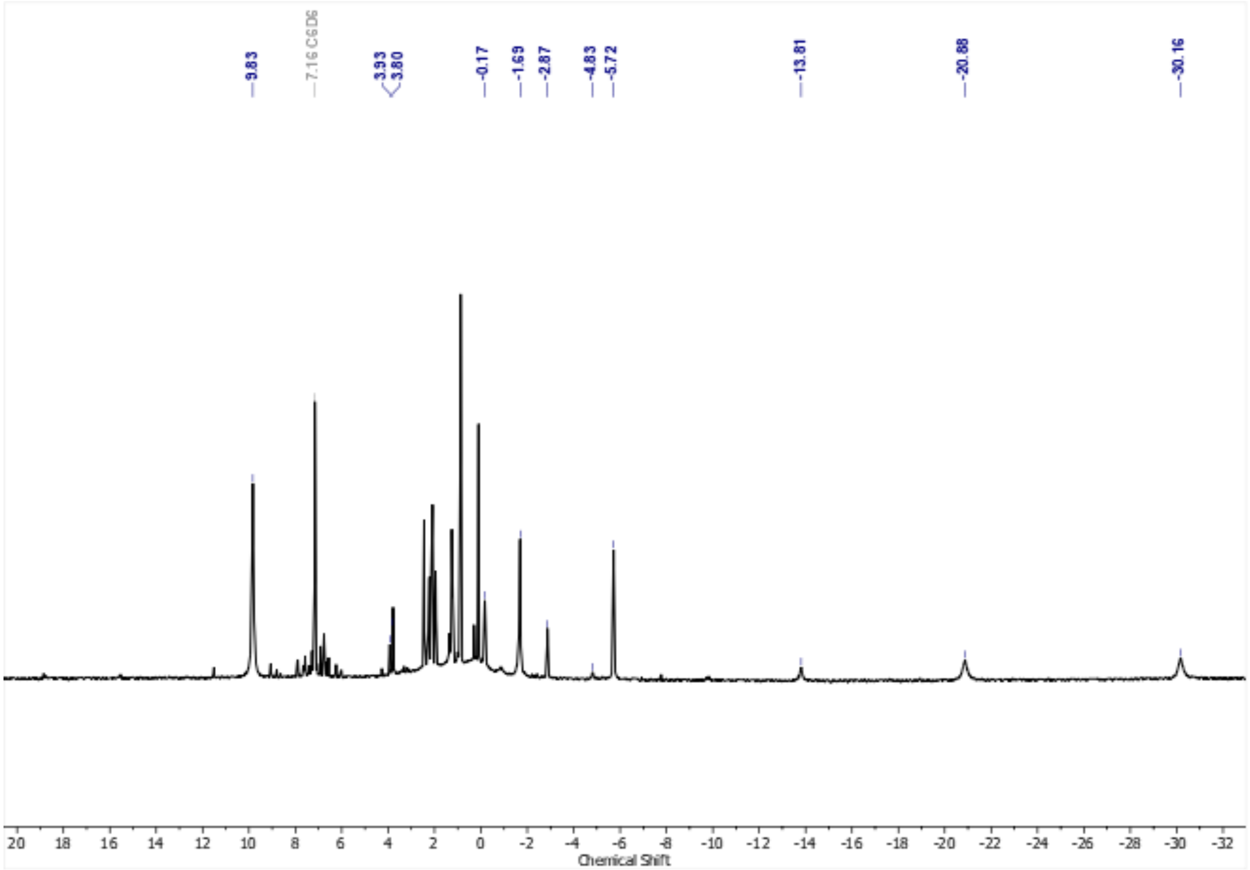


**Figure S1.** $^1$H NMR spectrum (400 MHz) of the attempt to synthesize U($^{Mes}$PDP$^{Ph}$)$_2$ via a one-pot salt metathesis reaction in benzene-d6. Note, formation of eleven major peaks is inconsistent with the expected number of resonances for the desired product.

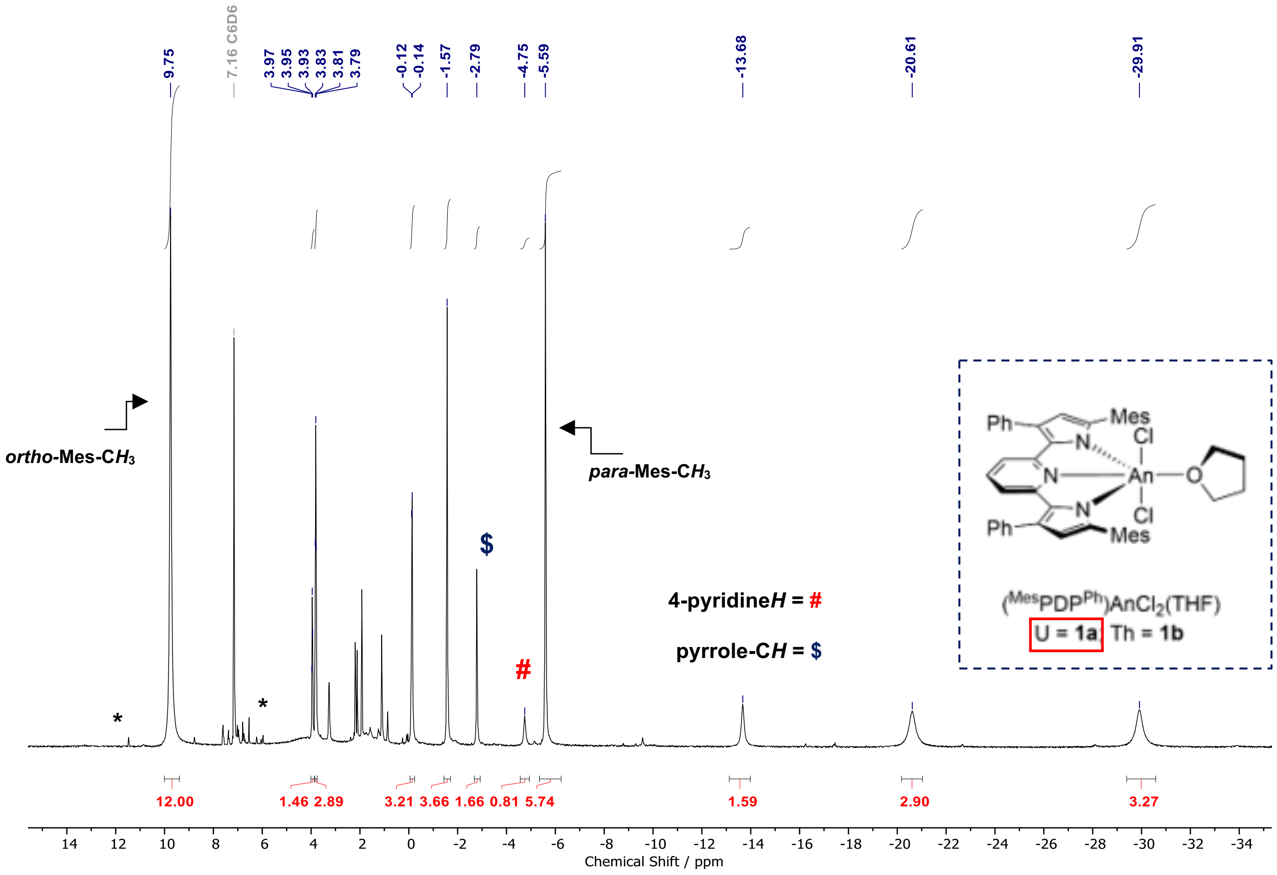


**Figure S2.** $^1$H NMR spectrum (400 MHz) of $^{Mes}PDP^{Ph}UCl_2(THF)$ (**1a**) in benzene-d6. Identifiable paramagnetic peaks with unique resonances are labeled; those that could not be formally assigned (2H and 4H resonances) were left unlabeled. The unmarked resonances correspond to toluene, pentane, and diethyl ether present in the sample and the starred (*) resonances correspond to a small amount of protonated ligand ($H_2{}^{Mes}PDP^{Ph}$) present in the sample.

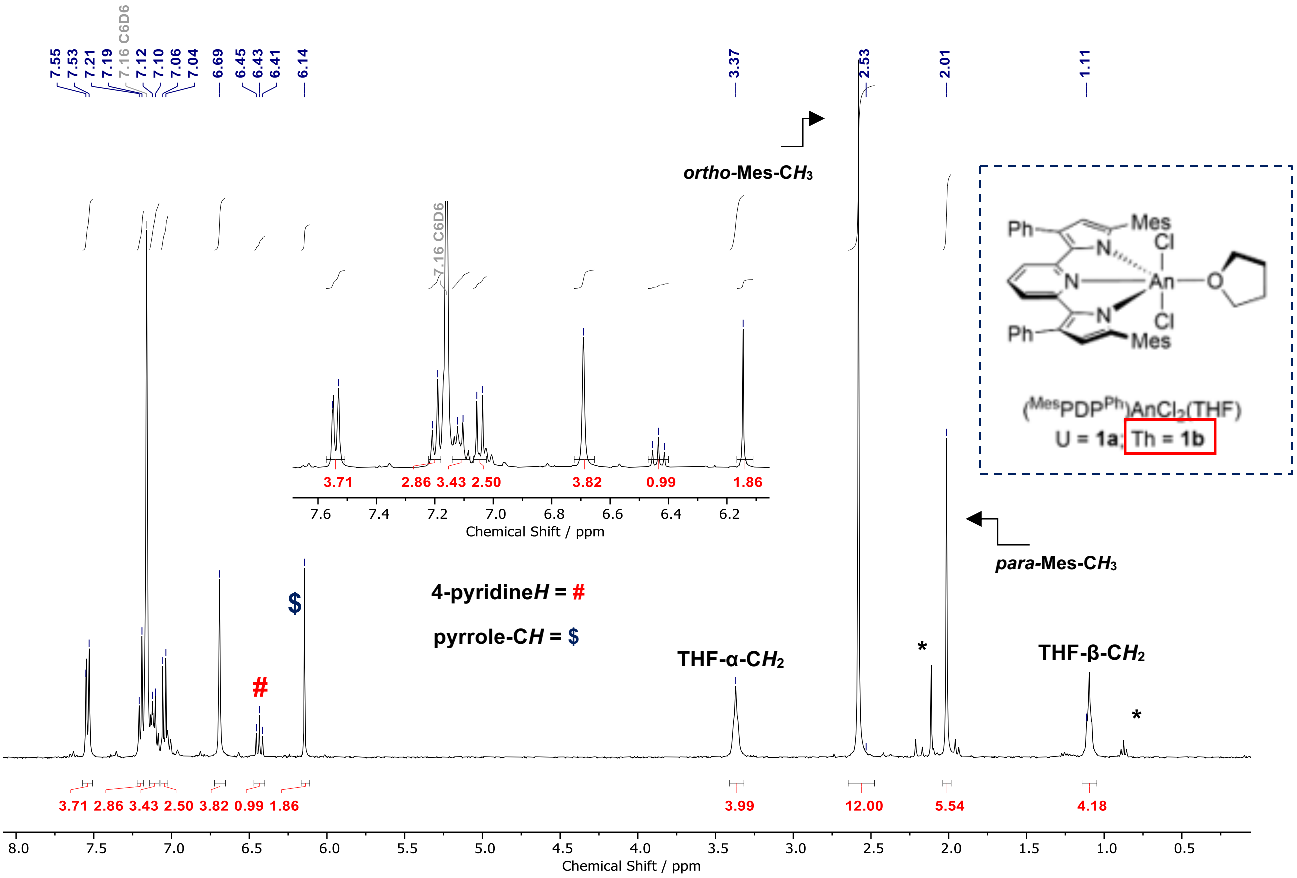


**Figure S3.** [1]H NMR spectrum (400 MHz) of $^{Mes}PDP^{Ph}ThCl_2(THF)$ (**1b**) in benzene-d6. Inset shows the aromatic region of the spectrum magnified to increase visibility of resonances in that region. Identifiable diamagnetic peaks with unique resonances are labeled; those that could not be formally assigned (2H and 4H resonances) were left unlabeled. The starred (*) resonances correspond to toluene and pentane present in the sample. Note, the resonances at 7.19 and 7.12 ppm are slightly overlapping with the signal for deuterated solvent.

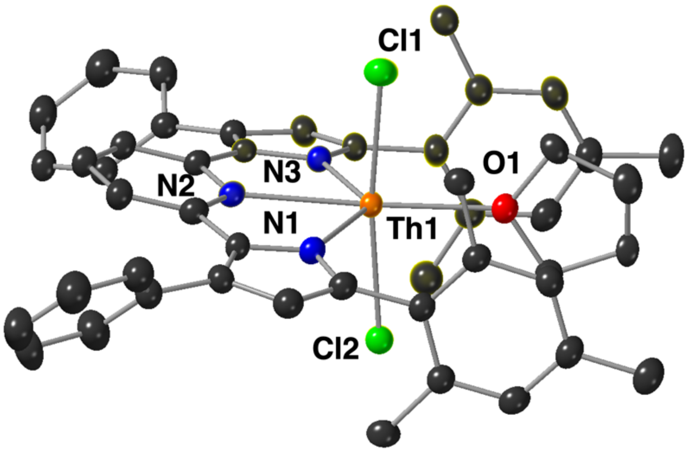


($^{Mes}$PDP$^{Ph}$)$ThCl_2$(THF) (**1b**)

**Figure S4.** Molecular structure of ($^{Mes}$PDP$^{Ph}$)$ThCl_2$(THF) (**1b**) shown with 30% probability ellipsoids. Hydrogen atoms and solvent molecules have been removed for clarity. Key: dark green, Th; blue, N; gray, C; light green, Cl; red, O. All non-carbon atoms have been labeled in the image.

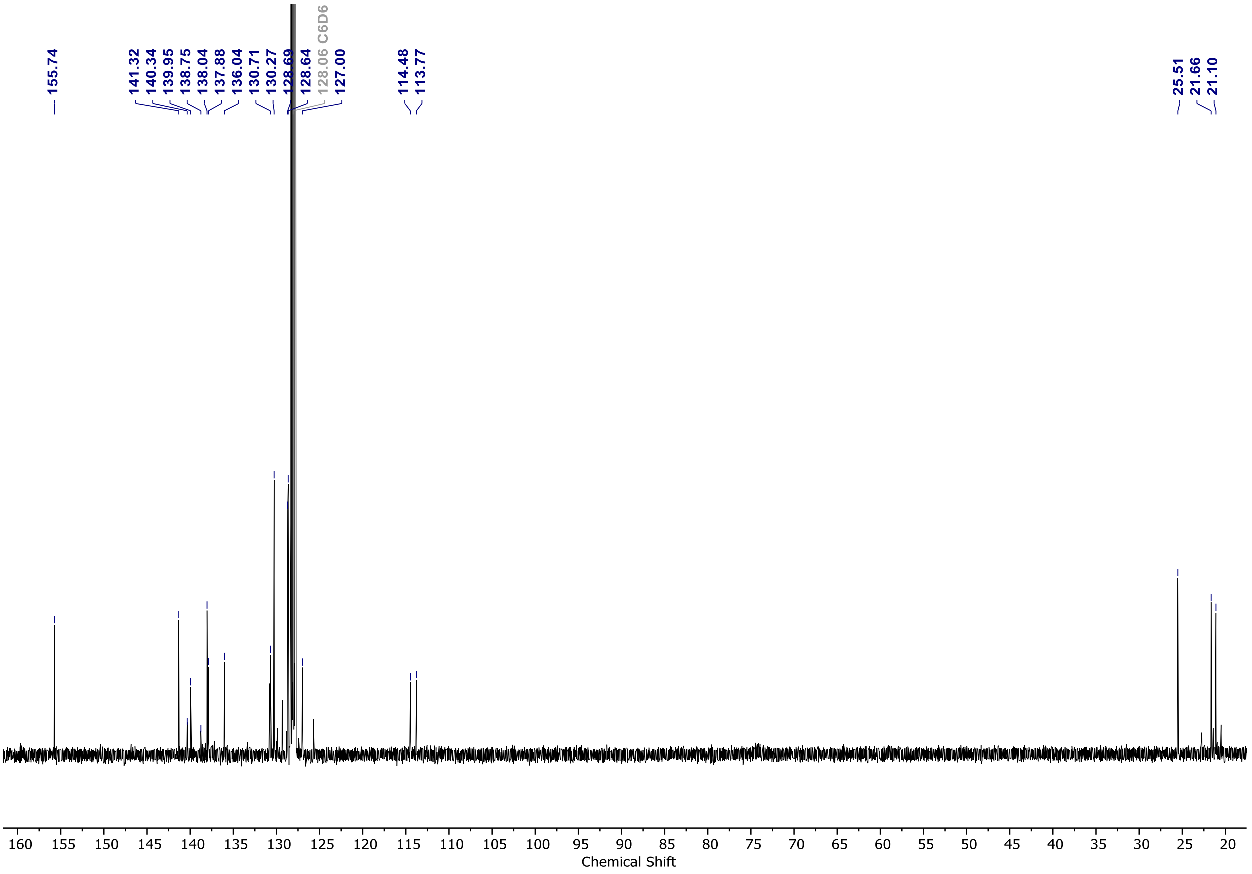


**Figure S5.** $^{13}C\{^1H\}$ NMR spectrum of $^{Mes}PDP^{Ph}ThCl_2(THF)$ (**1b**) in benzene-$d_6$.

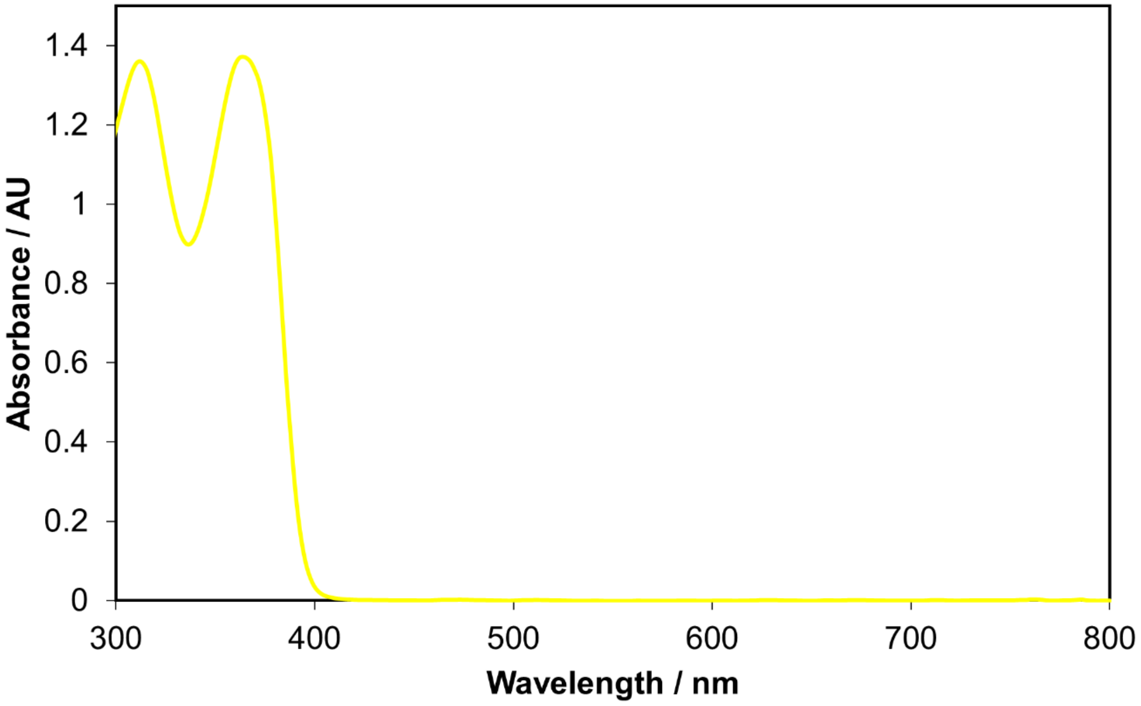


**Figure S6.** UV-Vis spectrum of $H_2{}^{Mes}PDP^{Ph}$ in anhydrous toluene at room temperature.

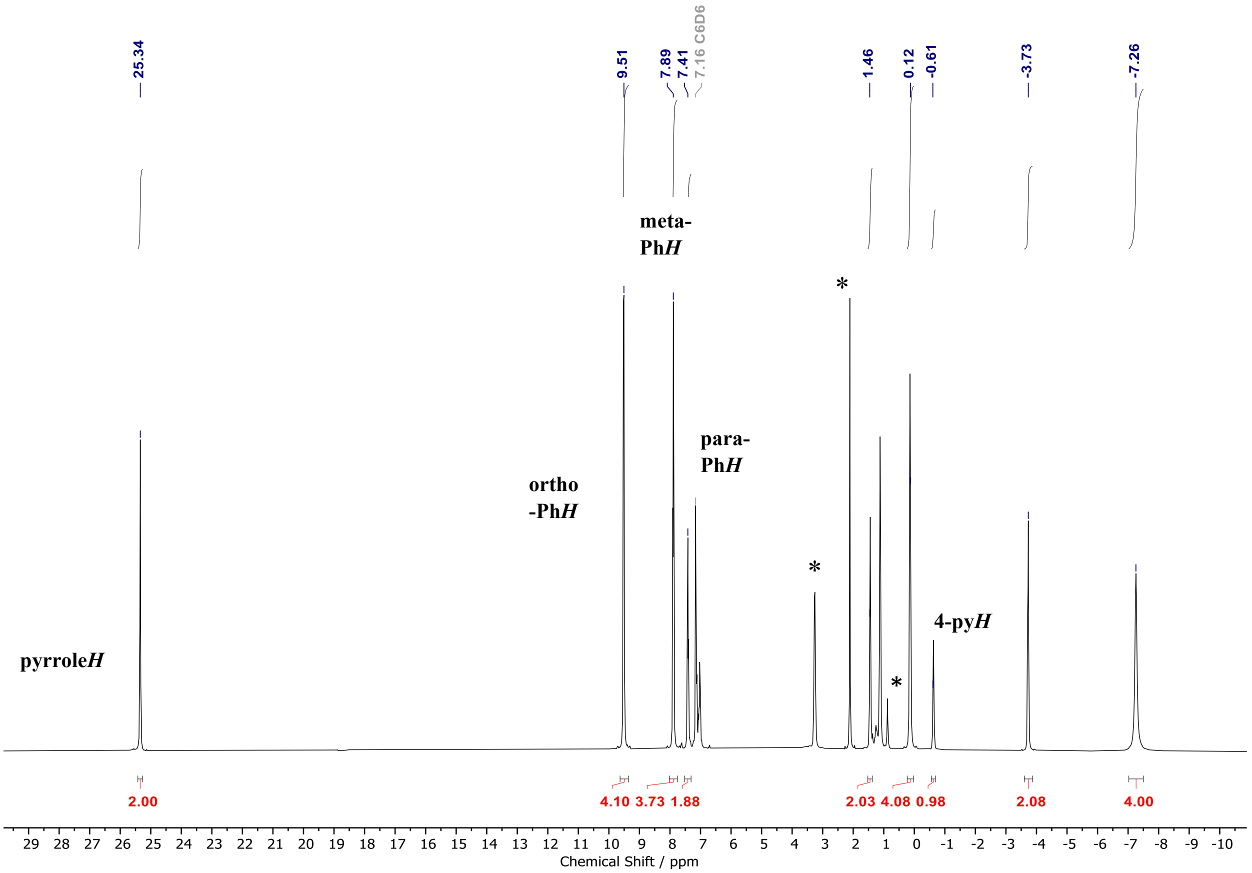


**Figure S7.** $^1$H NMR spectrum (400 MHz) of U($^{Ph}$PDP$^{Ph}$)$_2$ in benzene-d6. Unique resonances have been labeled. The starred (*) resonances correspond to toluene, pentane, and diethyl ether present in the sample.

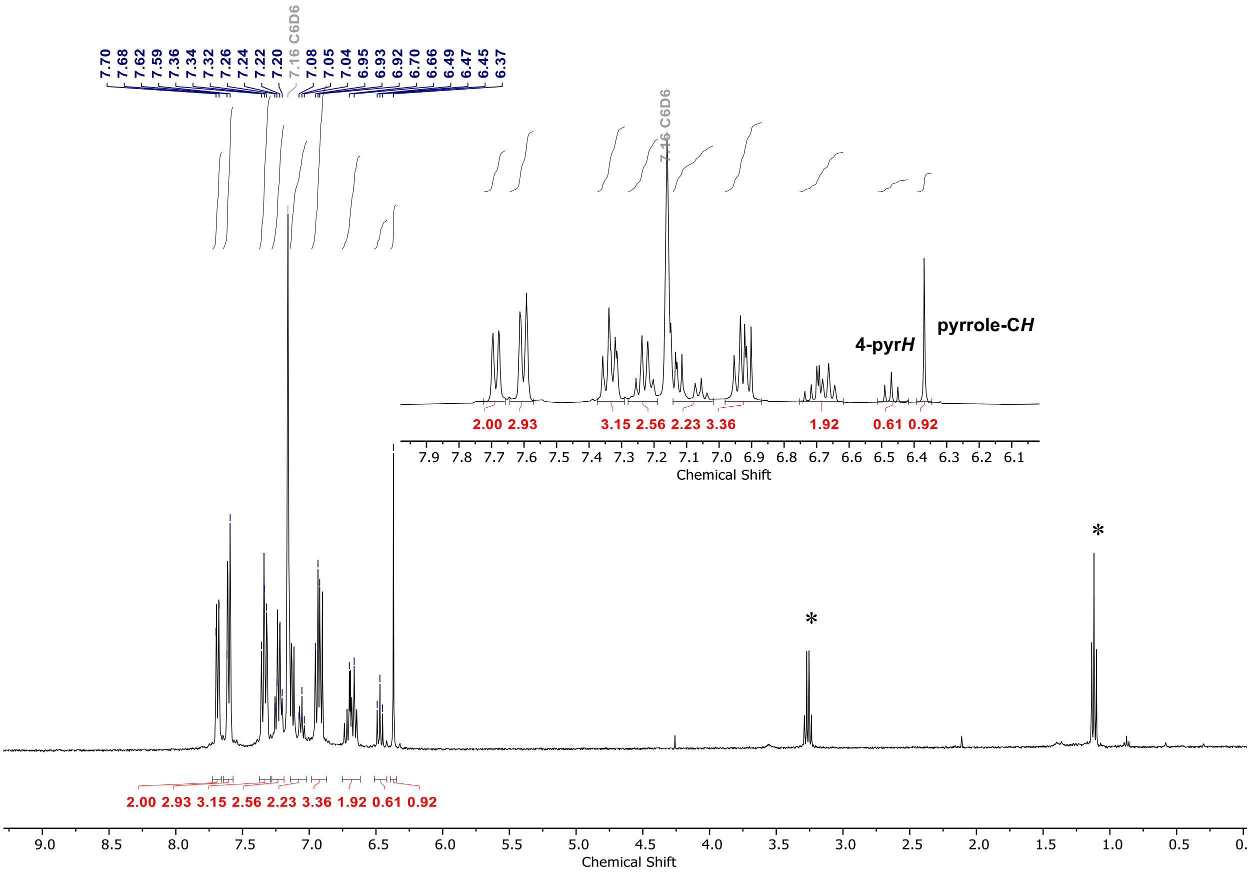


**Figure S8.** $^{1}$H NMR spectrum (400 MHz) of Th($^{Ph}$PDP$^{Ph}$)$_2$ in benzene-d6. Inset shows the aromatic region of the spectrum magnified to increase visibility of resonances in that region. Unique resonances are labeled. The starred (*) resonances correspond to diethyl ether present in the sample.

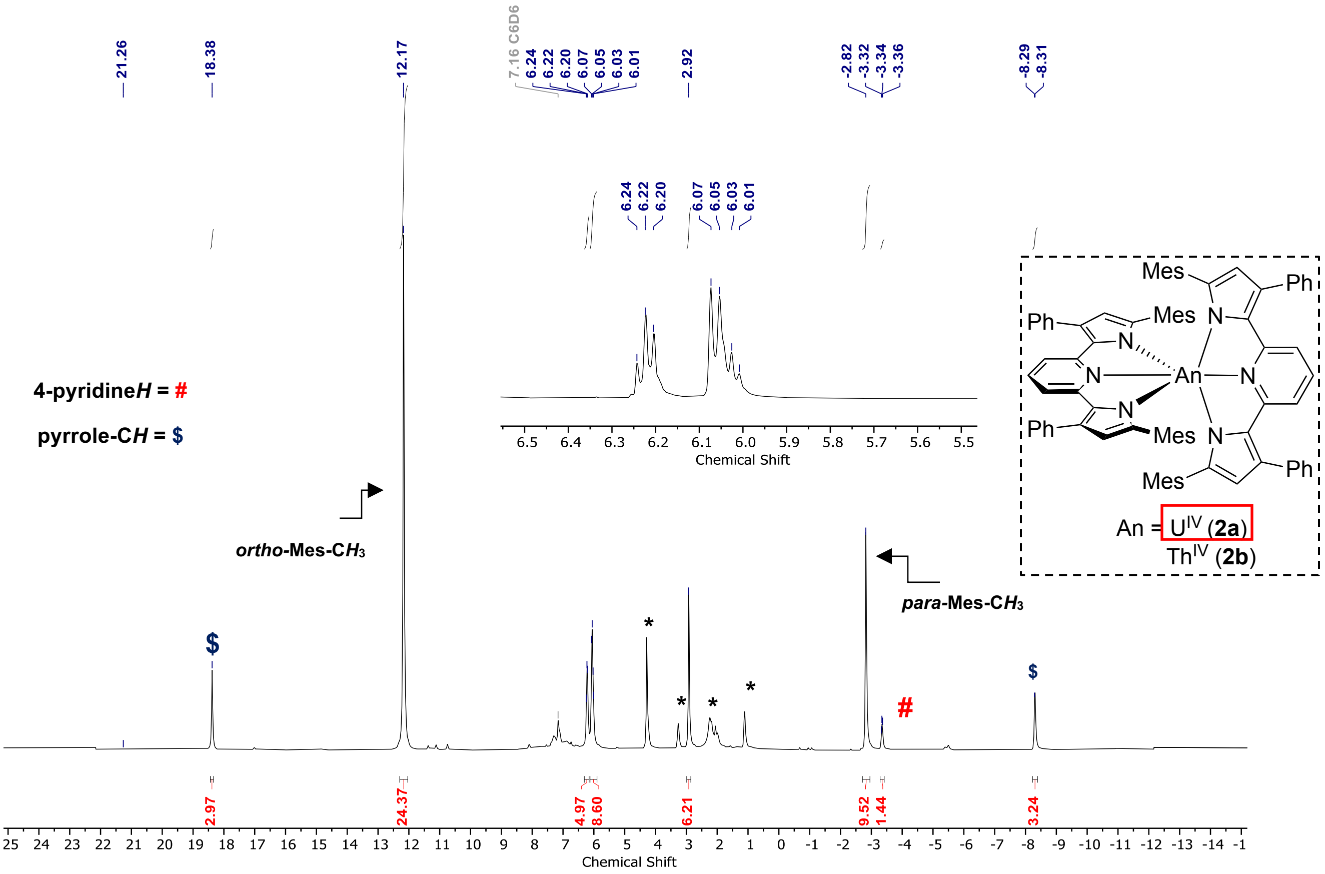


**Figure S9.** $^1$H NMR spectrum (400 MHz) of U($^{Mes}$PDP$^{Ph}$)$_2$ (**2a**) in benzene-d6. Identifiable paramagnetic peaks with unique resonances are labeled; those that could not be formally assigned (4 and 8 H resonances) were left unlabeled. The starred (*) resonances correspond to dichloromethane, pentane, and toluene present in the sample.

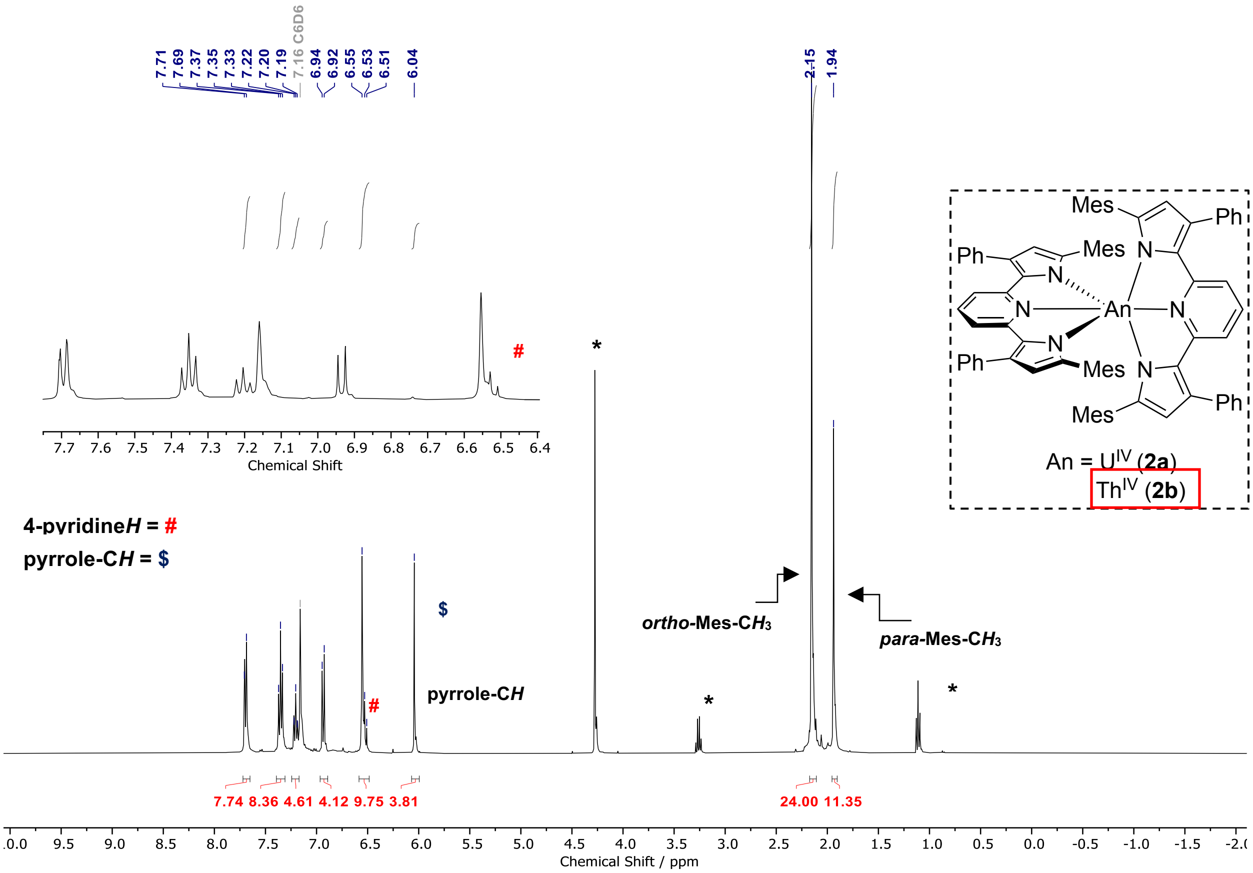


**Figure S10.** $^{1}$H NMR spectrum (400 MHz) of Th($^{Mes}$PDP$^{Ph}$)$_2$ (**2b**) in benzene-d6. Identifiable diamagnetic peaks with unique resonances are labeled; those that could not be formally assigned (4 and 8 H resonances) were left unlabeled. The starred (*) resonances correspond to dichloromethane and diethyl ether present in the sample.

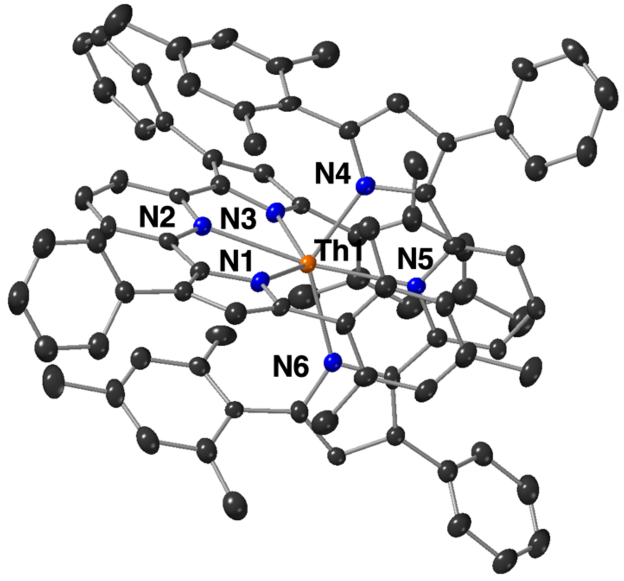


Th($^{Mes}$PDP$^{Ph}$)$_2$ (**2b**)

**Figure S11.** Molecular structure of Th($^{Mes}$PDP$^{Ph}$)$_2$ (**2b**) shown with 30% probability ellipsoids. Hydrogen atoms and solvent molecules have been removed for clarity. Key: orange, Th; blue, N; gray, C; light green, Cl; red, O. All non-carbon atoms have been labeled in the image.

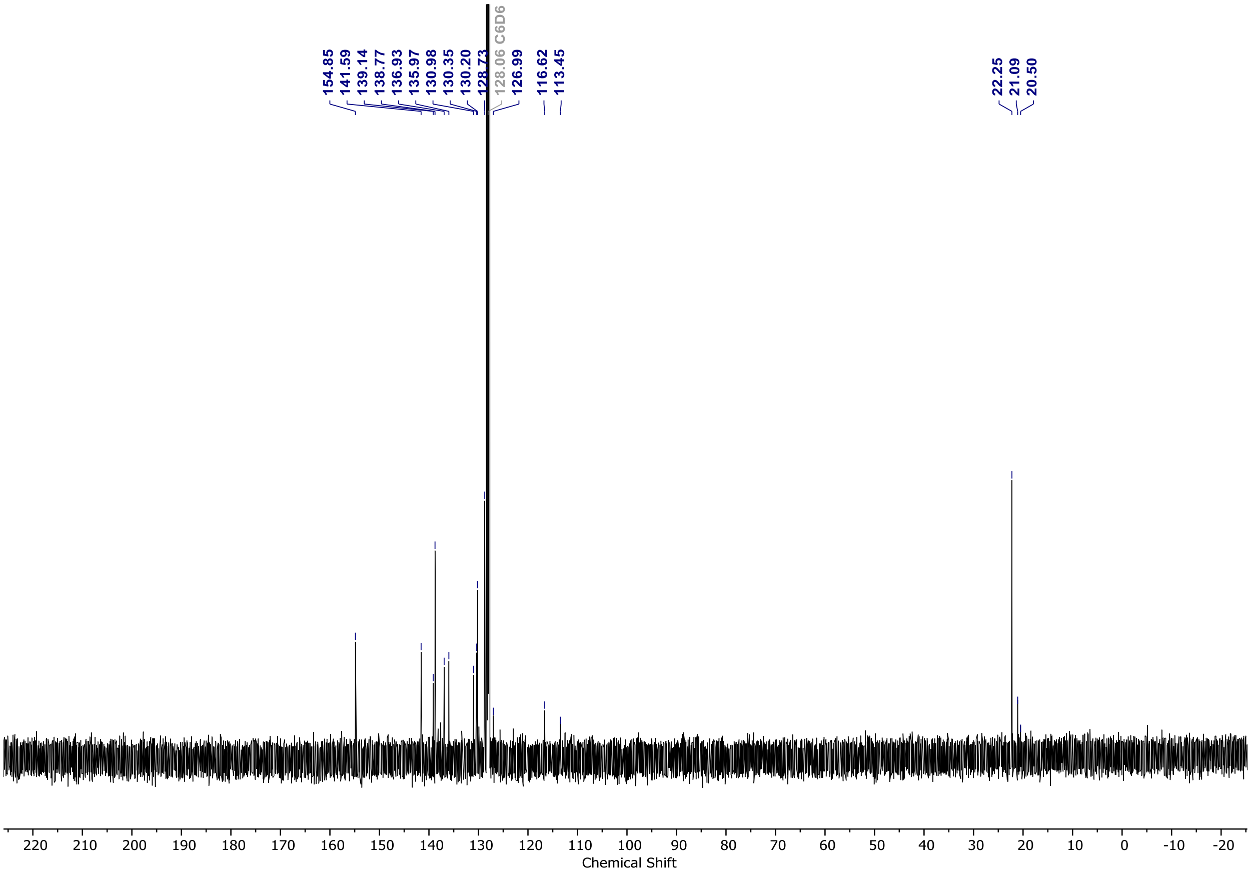


**Figure S12.** $^{13}C\{^{1}H\}$ NMR spectrum of Th($^{Mes}$PDP$^{Ph}$)$_2$ (**2b**) in benzene-d$_6$.

## Quantum Yield Determination

The quantum yields of **1b** and **2b** were determined via comparative method. Rhodamine 6G in ethanol (Φ = 0.95) was used as a standard. The quantum yield was calculated using the following equation:

$$\Phi_X = \Phi_Y * \left(\frac{Absorbance_Y}{Absorbance_X}\right)\left(\frac{\Sigma\ Emission_X}{\Sigma\ Emission_Y}\right)\left(\frac{\eta_X}{\eta_Y}\right)^2$$

The subscripts $X$ and $Y$ stand for the sample and reference, respectively. Φ is the photoluminescence quantum yield. η is the refractive index of the solvent.

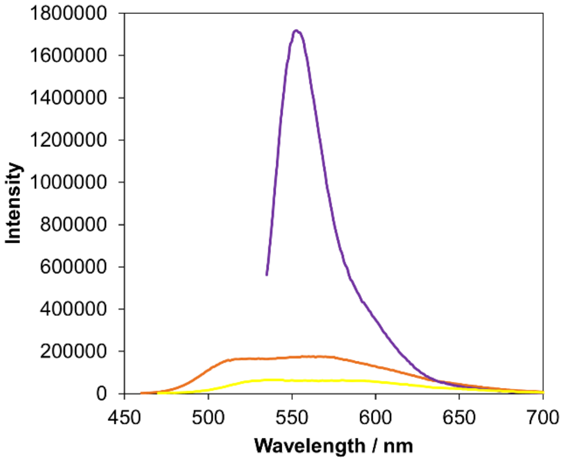


**Figure S13.** Emission of Rhodamine-6G in anhydrous ethanol plotted with **1b** and **2b** for determination of quantum yields via the comparative method.

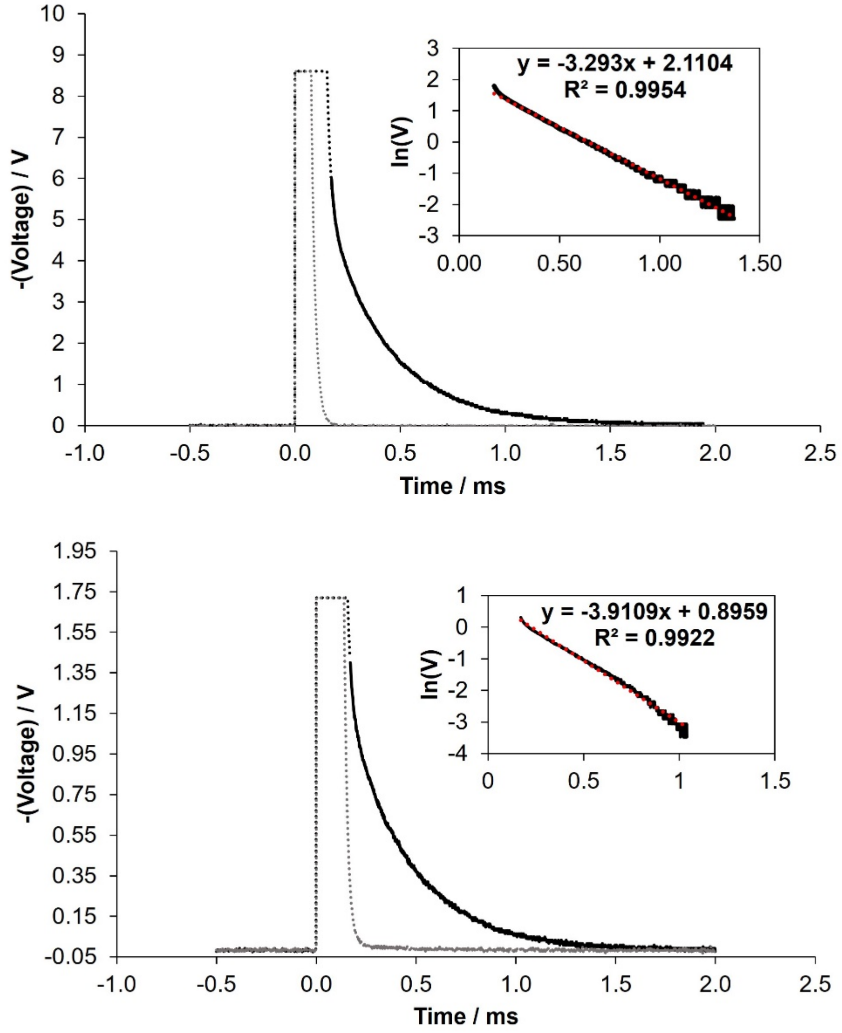


**Figure S14. Top:** Time profile for the phosphorescence intensity (304 μs) of **2b** at room temperature in toluene. Inset is time profile plotted on a logarithmic scale. **Bottom:** Time profile for the phosphorescence intensity (256 μs) of **1b** at room temperature in toluene. Inset is time profile plotted on a logarithmic scale.

**Input File Examples**
**1. Geometry Optimizations**

```
!rks pbe d3bj tightscf zora zora-def2-svp sarc/j normalprint tightopt

%pal nprocs 20 end

%maxcore 2000

%basis newgto Th "sarc-zora-tzvp" end
	newgto Cl "zora-def2-tzvp" end
	newgto O "zora-def2-tzvp" end
	newgto N "zora-def2-tzvp" end
end

*xyz 0 1
Coordinates from crystal structures
*
```

**2. TDDFT**

```
!rks b3lyp tightscf dkh dkh-def2-svp sarc/j normalprint cpcm(thf)

%pal nprocs 12 end

%maxcore 7000

%basis newgto Th "sarc-dkh-tzvp" end
	newgto Cl "dkh-def2-tzvp" end
	newgto O "dkh-def2-tzvp" end
	newgto N "dkh-def2-tzvp" end
end

%tddft nroots 70
	tda false
	donto true
	ntostates 1,2,3,4,5,6,7,8,9,10
	upop true
	irootlist 1,2,3,4,5,6,7,8,9,10
	trootlist 1,2,3,4,5,6,7,8,9,10
end

*xyz 0 1
Coordinates from geometry optimization
*
```
